# Sustainable Waste-Derived Mineral–Carbon Composites as Reusable Sorbents for Solid-Phase Extraction of Selected Organophosphorus Pesticides from Water

**DOI:** 10.3390/molecules31173082

**Published:** 2026-09-02

**Authors:** Piotr Słomkiewicz, Milena Górska, Dariusz Wideł, Julia Szewczuk

**Affiliations:** Institute of Chemistry, Jan Kochanowski University, 25-406 Kielce, Poland; piotr.slomkiewicz@ujk.edu.pl (P.S.); dariusz.widel@ujk.edu.pl (D.W.); julia.szewczuk54@gmail.com (J.S.)

**Keywords:** halloysite nanotubes, biochar, gas chromatography-tandem mass spectrometry (GC-MS/MS), trace analysis, agrochemicals, chlorpyrifos

## Abstract

Organophosphorus pesticides (OPs) are persistent environmental contaminants requiring monitoring in aquatic ecosystems. In this study, four novel mineral–carbon composites (K-HNT, K-C-HNT, D-HNT, and D-C-HNT) were synthesized via thermal treatment at 800 °C from halloysite nanotubes, casein, dextrin, and pyrolyzed sawdust. Moreover, these materials were characterized (BET, FT-IR, Raman, SEM/EDS), revealing high specific surface areas (up to 218.3 m^2^/g for the D-C-HNT composite), and evaluated as solid-phase extraction (SPE) sorbents for the determination of six OPs from water. The eluates were analyzed using GC-MS/MS in MRM mode. This study examined the effect of eluent, sorbent mass, and multiple extractions on SPE efficiency. Ethyl acetate-n-hexane (1:1, *v*/*v*) was confirmed to be the most effective eluent, achieving high pesticide recoveries (>80%) at optimal sorbent masses of 50 and 75 mg. The method demonstrated satisfactory sensitivity, with limits of detection (LODs) ranging from 1.6 to 3.3 μgL^−1^. Notably, the synthesized composites outperformed pure precursors and demonstrated extraction efficiency comparable to commercial sorbents (C-18, Florisil, active carbon). Furthermore, reusability tests confirmed that second and third extraction repetitions reduced the efficiency of the SPE process only slightly, indicating that these waste-derived composites can be reused multiple times without cartridge replacement.

## 1. Introduction

Organophosphate pesticides (OPs) are commonly applied agrochemicals used to improve agricultural productivity and control a broad spectrum of insect pests. Despite their effectiveness, these compounds are characterized by high toxicity and adverse effects on living organisms [1,2]. Concerns have been raised regarding their persistence in the environment and potential risks to human health. OPs can contaminate soil, water, and food products, leading to dietary and occupational exposure [1,2,3], and their neurotoxic effects are well documented [4,5,6]. Their primary toxicological mechanism is the inhibition of acetylcholinesterase, leading to the accumulation of acetylcholine and overstimulation of the nervous system, which can cause respiratory failure and death [4]. Beyond acute effects, chronic exposure to OPs has been associated with endocrine disruption, neurodevelopmental deficits, and reproductive impairments, including reduced fertility, impaired embryonic development, and hormonal imbalances [3,5,6,7,8]. Several OPs, including chlorpyrifos, diazinon, and parathion, are frequently detected in food and environmental samples and are listed as priority pollutants due to their widespread use, persistence, and documented health risks [9,10,11,12,13,14,15]. Consequently, monitoring of environmental and food samples is essential to ensure compliance with international regulations, including EU and World Health Organization guidelines. According to EU Commission Regulation 2020/1085 of 23 July 2020, chlorpyrifos and chlorpyrifos-methyl are banned throughout the European Union, and the maximum residue level (MRL) was lowered to 0.01 mg/kg for all food and animal feed. Although these substances are now banned in the EU [16], they remain widely used in countries not subject to these regulations, meaning that imported food commodities from such regions may still contain pesticide residues. For this reason, testing for trace amounts of these pesticides in food products is necessary. The maximum concentration level of organophosphate pesticides (OPs) for drinking water in European Union is set at 0.1 μg L^−1^ for individual compounds and 0.5 μg L^−1^ for total amount of pesticides [17].

Current removal strategies include advanced oxidation processes (AOPs), biodegradation, membrane filtration, and adsorption. Among these, adsorption is highly favored due to its simplicity, low cost, and high efficiency. A wide range of adsorbents has been investigated for the remediation of OPs from contaminated waters, including activated carbons, zeolites, biochars, metal-organic frameworks (MOFs), and various clay minerals. The successful application of these materials in environmental remediation inspires the development of miniaturized, high-performance sorbents for analytical sample preparation.

Due to the low concentration, the analytical methods used to determine the trace levels of pesticides in water may require an additional step with the concentration of the sample. The most used analytical methods for the detection of organophosphate pesticides are gas chromatography (GC), liquid chromatography (LC), high-performance liquid chromatography (HPLC), and their combinations with mass spectrometry (GC-MS, LC-MS) [18,19]. These techniques are often coupled with sample pre-concentration procedures to enable the effective isolation and detection of trace contaminants in water. Commonly applied methods include solid-phase extraction (SPE) [20], solid-phase microextraction (SPME) [21,22], microextraction by packed sorbent (MEPS) [23], microwave-assisted extraction (MAE) [24], magnetic solid-phase extraction (MSPE) [25], and liquid–liquid extraction (LLE) [26]. The choice of extraction method depends on the physicochemical properties of the target pesticides, the complexity of the matrix, and the analytical requirements.

Nevertheless, the traditional SPE method remains widely used, with ongoing efforts to find new adsorptive materials for packing analytical columns. The choice of sorbent affects both the efficiency and selectivity of the extraction. For organophosphorus pesticides, the most applied sorbents include C18, Florisil, graphene, multi-walled carbon nanotubes, and various polymers [27,28,29,30,31,32,33,34]. In recent years, research has also focused on mineral–carbon composites. These materials consist of a mineral component (e.g., kaolinite, zeolites, clays) combined with a carbon part (e.g., activated carbon, biochar). Unlike conventional solutions, such composites are often produced from waste materials (e.g., biomass or mineral residues), thereby supporting the ideas of sustainable development and circular economy. Due to well-developed specific surface area, a homogeneous micro- and mesoporous structure, and numerous active sites, they are suitable for removing a wide variety of contaminants. Moreover, these materials are more durable and mechanically resistant than conventional sorbents like C18 and Florisil because of the mineral component. Mineral–carbon composites have already been applied to the adsorption of heavy metals, phthalates, dyes, and pharmaceuticals [35]. In this study, the focus is placed on organophosphorus pesticides, as no similar investigations have been reported.

The main goal of this study was to evaluate the potential application of the following mineral–carbon composites (K-HNT, D-HNT, K-C-HNT, and D-C-HNT) as sorbents in the solid-phase extraction of selected organophosphate pesticides (chlorpyrifos, disulfoton, ethoprofos, fenchlorfos, parathion-methyl, and prothiofos) from water. The qualitative and quantitative determination of these pesticides was carried out using GC-MS/MS. A novelty in the research was the use of two-component sorbents synthesized from casein or dextrin and halloysite nanotubes (HNTs), as well as three-component materials enriched with pyrolyzed pine sawdust as an additional source of carbon. To our knowledge, materials of this complexity have not been reported previously, which motivated the present investigation.

## 2. Results and Discussion

### 2.1. Mineral–Carbon Composite Properties

**Figure 1:**
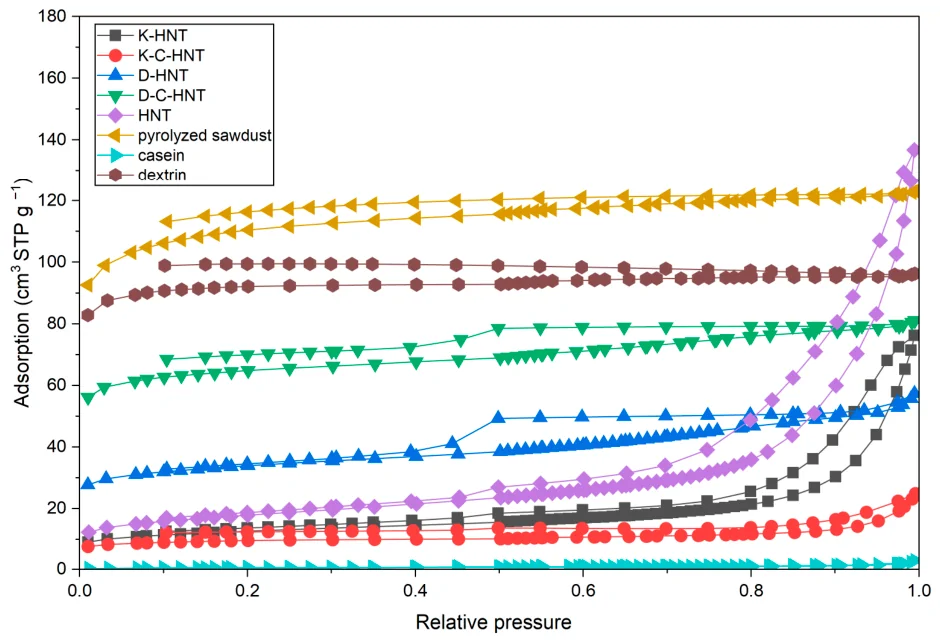
Nitrogen adsorption-desorption isotherms of raw HNT, carbon precursors, and the synthesized mineral–carbon composites.

The nitrogen adsorption–desorption isotherms of the raw materials and prepared composites are illustrated in Figure 1, and the obtained structural surface parameters are presented in Table 1. For the composites, the highest specific surface area (S_BET_) was observed for the dextrin-based materials, including D-HNT (115.6 m^2^ g^−1^) and D-C-HNT (218.25 m^2^ g^−1^). In contrast, the casein-based materials showed lower specific surface area values, as presented for K-HNT (44.15 m^2^ g^−1^) and K-C-HNT (32.80 m^2^ g^−1^). The next evaluated parameter was the total pore volume (V_t_). The highest values were recorded for D-C-HNT (0.1245 cm^3^ g^−1^) and K-HNT (0.1105 cm^3^ g^−1^). The K-HNT composite was characterized by the highest mesoporosity (93.21%), while the D-C-HNT composite, which was a microporous material, had the lowest mesoporosity (36.06%). As for the precursor materials, pyrolyzed sawdust features a high specific surface area (351.06 m^2^/g) and is predominantly microporous. In contrast, halloysite nanotubes (HNTs) have a lower surface area (62.67 m^2^/g) but are highly mesoporous (95.86%), with a mesopore volume of 0.1877 cm^3^/g. Regarding the casein-derived carbon material, it exhibits the lowest specific surface area, equal to only 1.86 m^2^/g. As observed for the composites, the addition of casein led to a decrease in S_BET_ and an increase in mesoporosity, which may suggest partial blocking of micropores. In contrast, dextrin shows a relatively high S_BET_ of 308.01 m^2^/g, and all dextrin-based composites display higher S_BET_ values and a more microporous character.

### 2.2. FT-IR Spectroscopy

**Figure 2:**
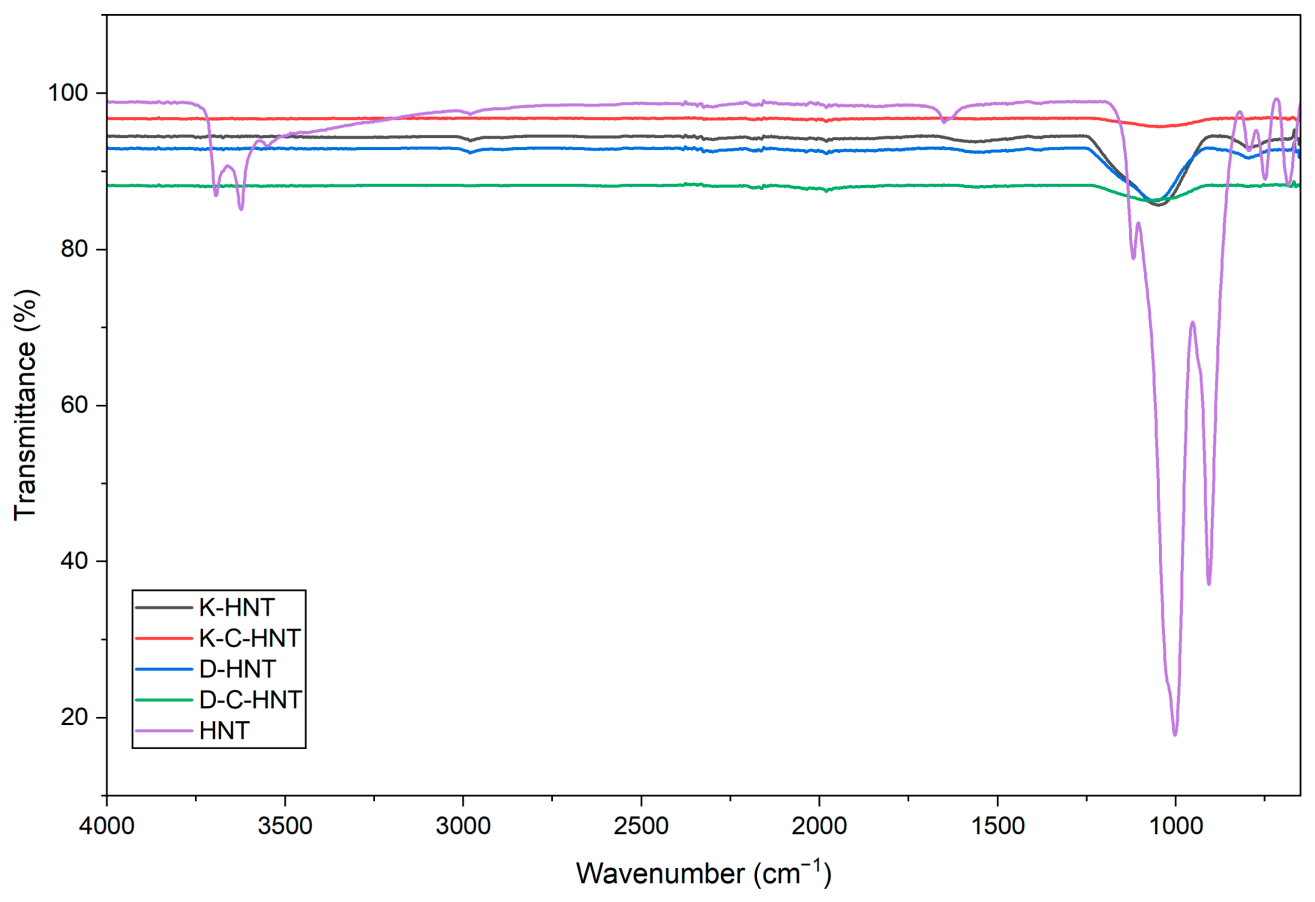
FT-IR spectra of studied composites and HNT in the range of 4000–650 cm^−1^.

Figure 2 displays the FT-IR spectra of unmodified halloysite nanotubes (HNTs) alongside the synthesized mineral–carbon composites (K-HNT, K-C-HNT, D-HNT, and D-C-HNT) across the wavenumber range of 4000 to 650 cm^−1^. The spectrum of pure HNT exhibits intense, well-defined absorption bands, most notably featuring sharp double peaks in the hydroxyl stretching region between 3700 and 3600 cm^−1^. The absorption bands observed at approximately 3690 cm^−1^ and 3620 cm^−1^ correspond to the stretching vibrations of inner-surface hydroxyl groups (Al–OH) situated at the octahedral interface and inner hydroxyl groups within the nanotube lumen, respectively.

Additionally, pure HNT displays a very deep, prominent absorption complex in the lattice vibration region between 1100 and 900 cm^−1^, where transmittance drops significantly to approximately 18–38%. The intense absorption band centered around 1030 cm^−1^ (with a characteristic shoulder near 1100 cm^−1^) is assigned to (Si–O–Si) stretching vibrations of the tetrahedral sheet, while the sharp band at 910 cm^−1^ corresponds to the (Al–O–H) bending vibration.

In contrast, the spectra for all four composite materials (K-HNT, K-C-HNT, D-HNT, and D-C-HNT) appear as nearly flat, horizontal lines grouped tightly within the 88% to 97% transmittance range. Upon incorporating the carbonized precursors, the characteristic mineral peaks observed in pure HNTs become almost completely masked. This visual flattening across both the hydroxyl and lattice vibration regions confirms that the HNTs have been successfully covered by a continuous layer of a carbonized part.

The absorption bands observed for pure HNTs at 3690, 3620, 1030, and 910 cm^−1^ are consistent with the vibrational modes of halloysite reported in previous studies [36,37]. The visual flattening of the spectra across both the hydroxyl and lattice vibration regions for the synthesized composites is a characteristic feature of carbon-coated minerals. As noted in the literature regarding polymer and carbon-coated HNTs [37], this masking effect occurs because the amorphous carbon layer strongly absorbs infrared radiation, effectively obscuring the underlying mineral signals.

### 2.3. Raman Spectroscopy

**Figure 3:**
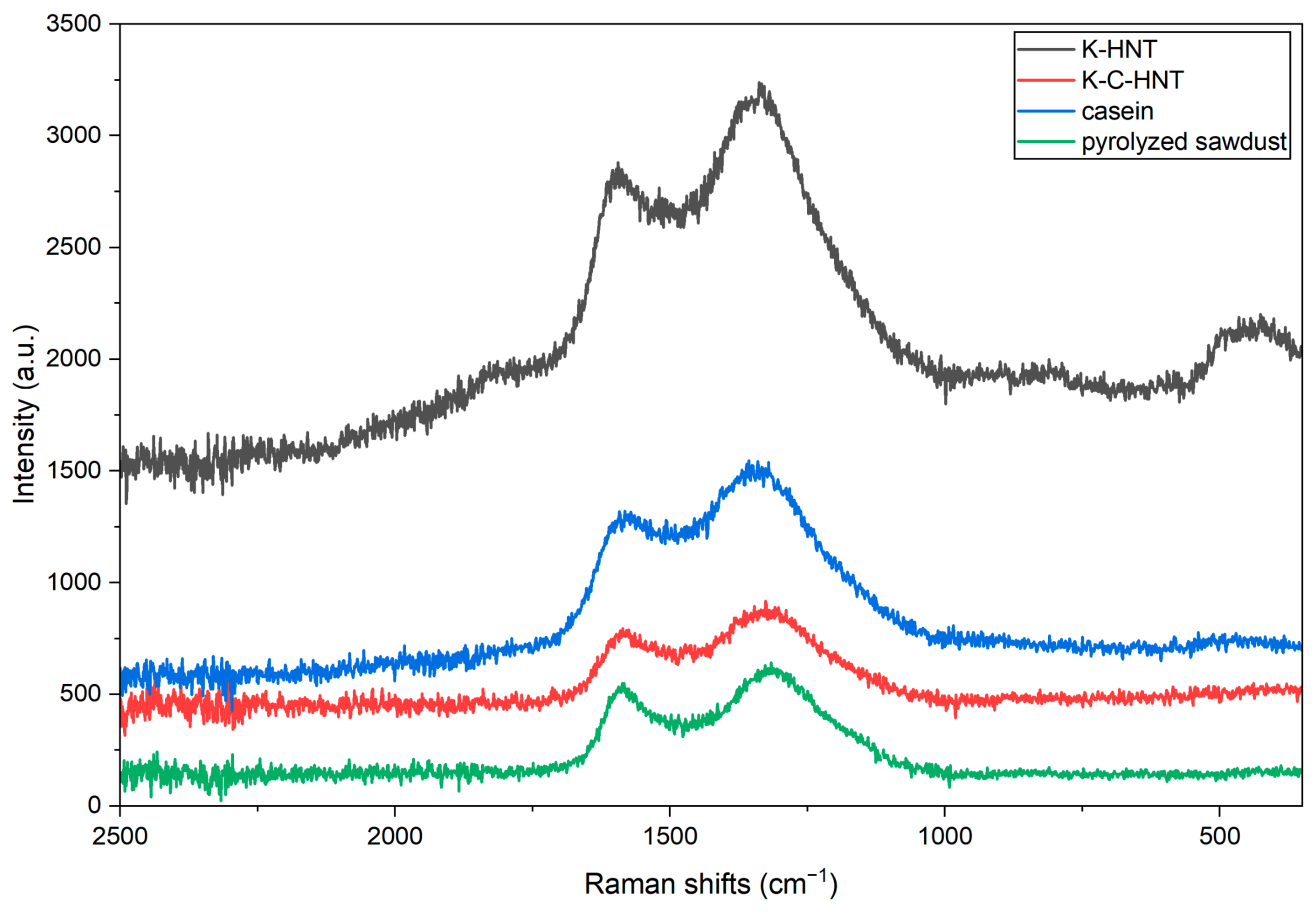
Raman spectra of casein-based composites with their pure organic precursors measured in the range of 2500–450 cm^−1^.

**Figure 4:**
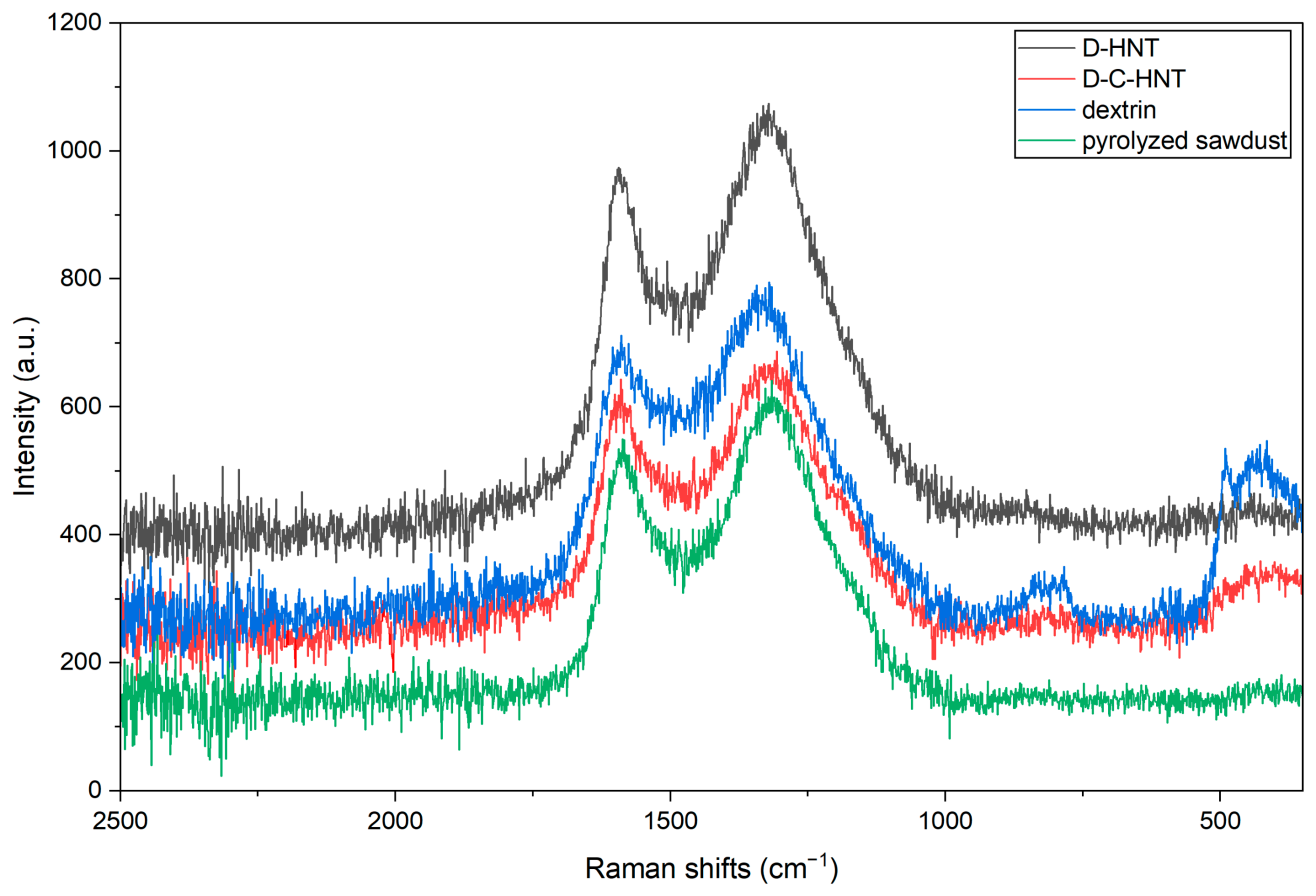
Raman spectra of dextrin-based composites with their pure organic precursors measured in the range of 2500–450 cm^−1^.

Figure 3 and Figure 4 display the Raman spectra of the casein-based (K-HNT and K-C-HNT) and dextrin-based (D-HNT and D-C-HNT) composites, along with their pure organic precursors, measured across the 2500–450 cm^−1^ shift range. Across all analyzed samples, the spectra exhibit two prominent characteristics of carbonaceous materials: the D-band centered near 1350 cm^−1^ and the G-band near 1585 cm^−1^. The pronounced linewidths between these two bands confirm that the carbon coatings formed during pyrolysis possess a turbostratic, disordered architecture rather than an ordered crystalline graphitic lattice.

The degree of carbon graphitization (R) was calculated using Equation (1), and the crystallinity of carbon was determined using Equation (2).

(1) $$R=\frac{I_{D}}{I_{G}}$$

(2) $$K=\frac{I_{G}}{I_{G}+I_{D}}\cdot 100\%$$

where I_G_—integrated intensity of the G-band [cm^−1^], I_D_—integrated intensity of the D-band [cm^−1^]; K—crystallinity coefficient [%].

The values of the R parameter and the crystallinity coefficients for the studied composites and the carbon materials are presented in Table 2.

As shown in Table 2, the quantitative Raman spectroscopy results demonstrate similarity across all evaluated precursors and mineral–carbon composites. Specifically, the defect parameter (R) falls within a very narrow range of 0.834 to 0.848, while the crystallinity coefficient (K) remains between 54.100% and 54.515% across all samples. Overall, these highly similar values confirm that a stable, reproducible turbostratic carbon architecture is formed during pyrolysis.

The positions of the D-band (defect-induced, ~1350 cm^−1^) and G-band (graphitic, ~1585 cm^−1^) are typical for turbostratic, disordered carbonaceous materials and align with Raman spectra reported for biomass-derived carbons [38]. Furthermore, the calculated defect parameters confirm that the carbon consists primarily of small graphitic domains embedded in an amorphous matrix, which is consistent with the structure of cellulosic biochars [38]. This architecture is beneficial for adsorption processes due to the abundance of active edge sites.

### 2.4. SEM/EDS Analysis

**Figure 5:**
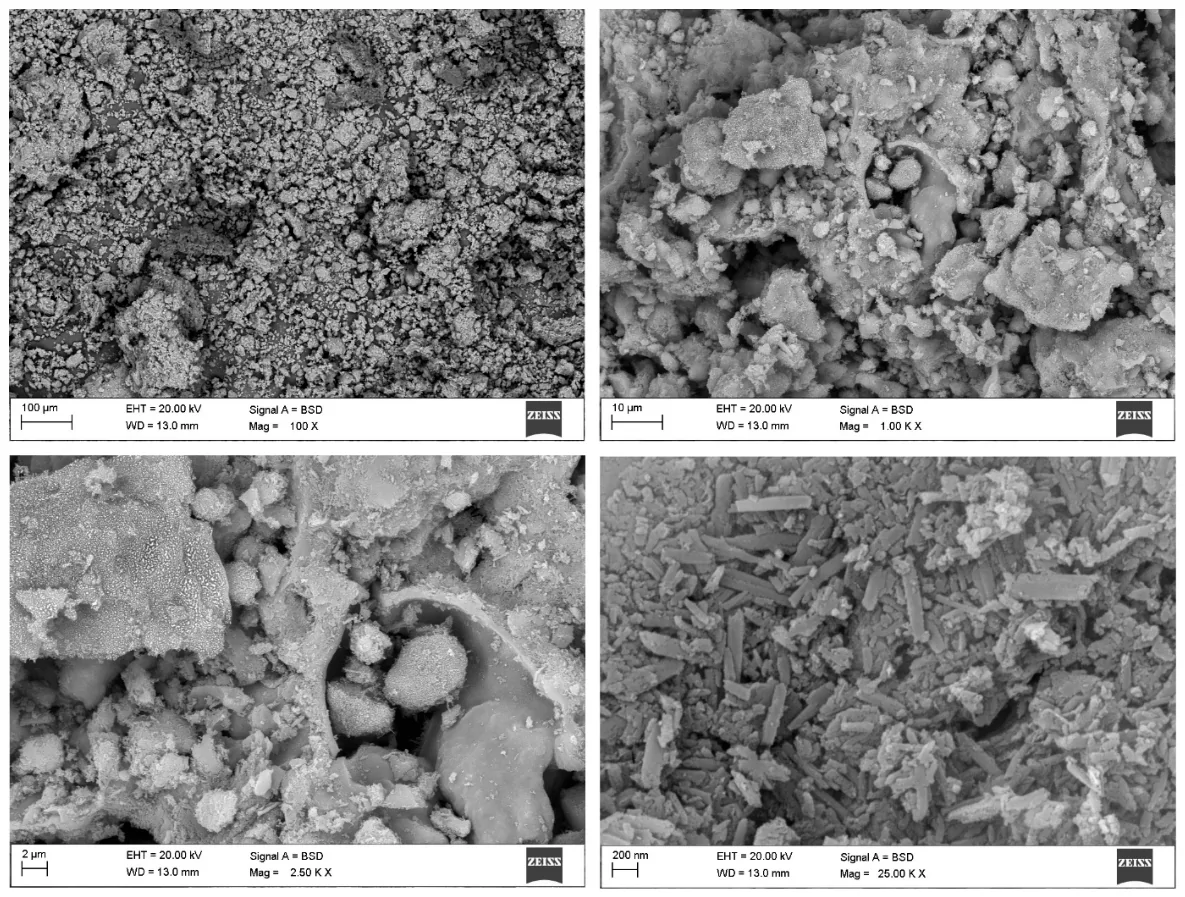
SEM images of studied mineral–carbon composite (K-HNT), magnification 100×, 1000×, 2500×, 25,000×.

**Figure 6:**
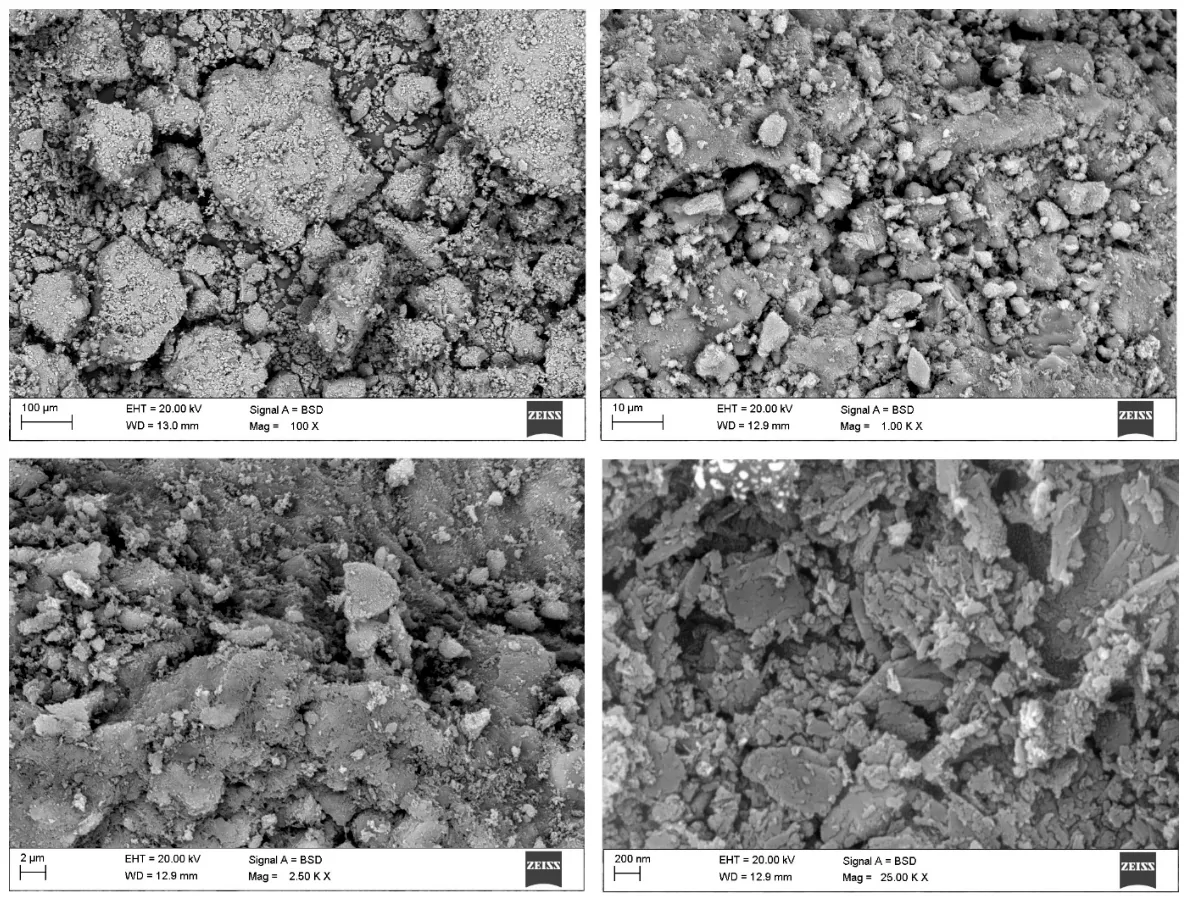
SEM images of studied mineral–carbon composite (D-HNT), magnification 100×, 1000×, 2500×, 25,000×.

**Figure 7:**
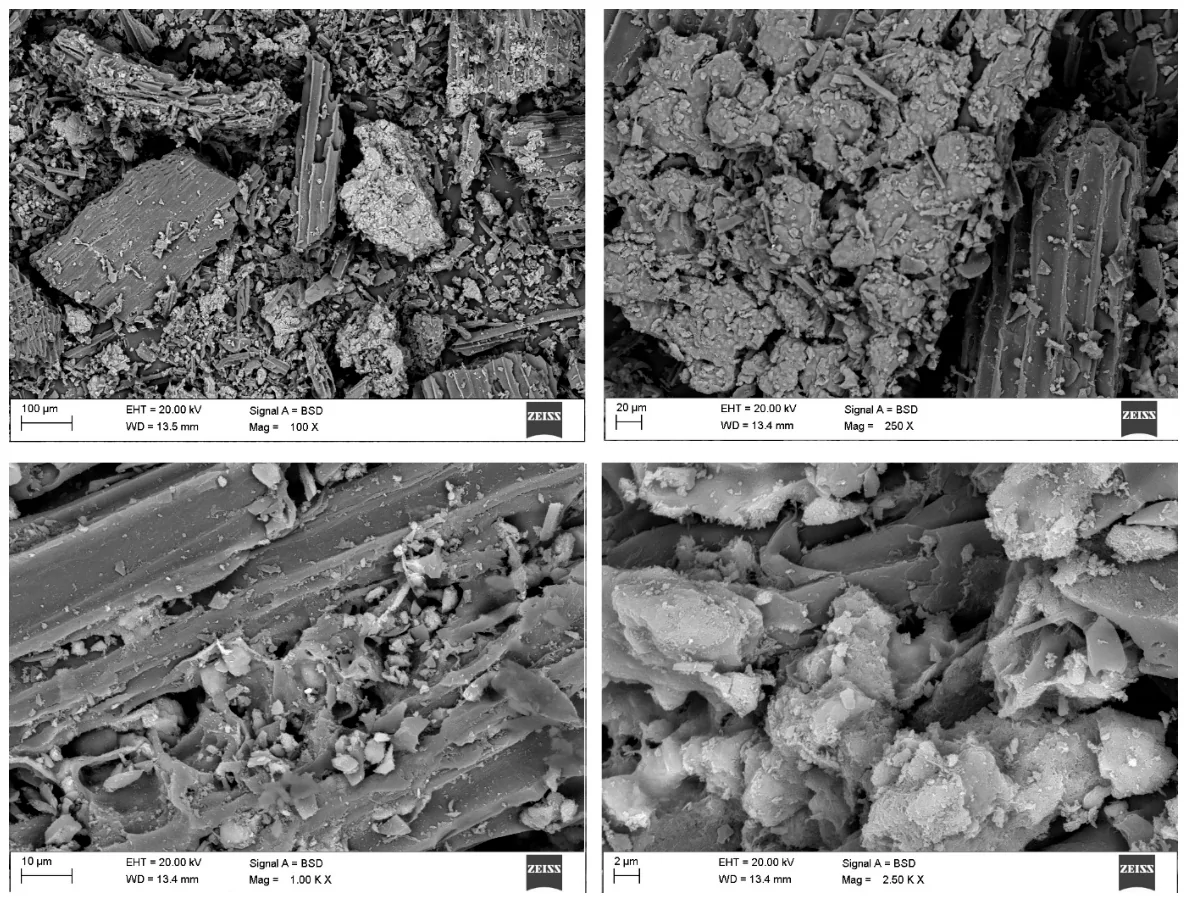
SEM images of studied mineral–carbon composite (K-C-HNT), magnification 100×, 250×, 1000×, 2500×.

The surface morphology of four mineral–carbon composites was evaluated using scanning electron microscopy (SEM) and is presented in Figure 5, Figure 6 and Figure 7. SEM images for the K-HNT and D-HNT composites are shown in Figure 5 and Figure 6, respectively. At lower magnifications ranging from 100× to 1000×, both materials exhibit a highly agglomerated, irregular granular structure. The amorphous carbon matrices, derived from the carbonization of the casein and dextrin binders, act as a phase that encapsulates the mineral particles. At a higher magnification of 25,000×, the characteristic tubular morphology of the halloysite nanotubes (HNTs) becomes visible in both samples. The HNTs are densely packed and randomly oriented within the carbonized matrices.

**Figure 8:**
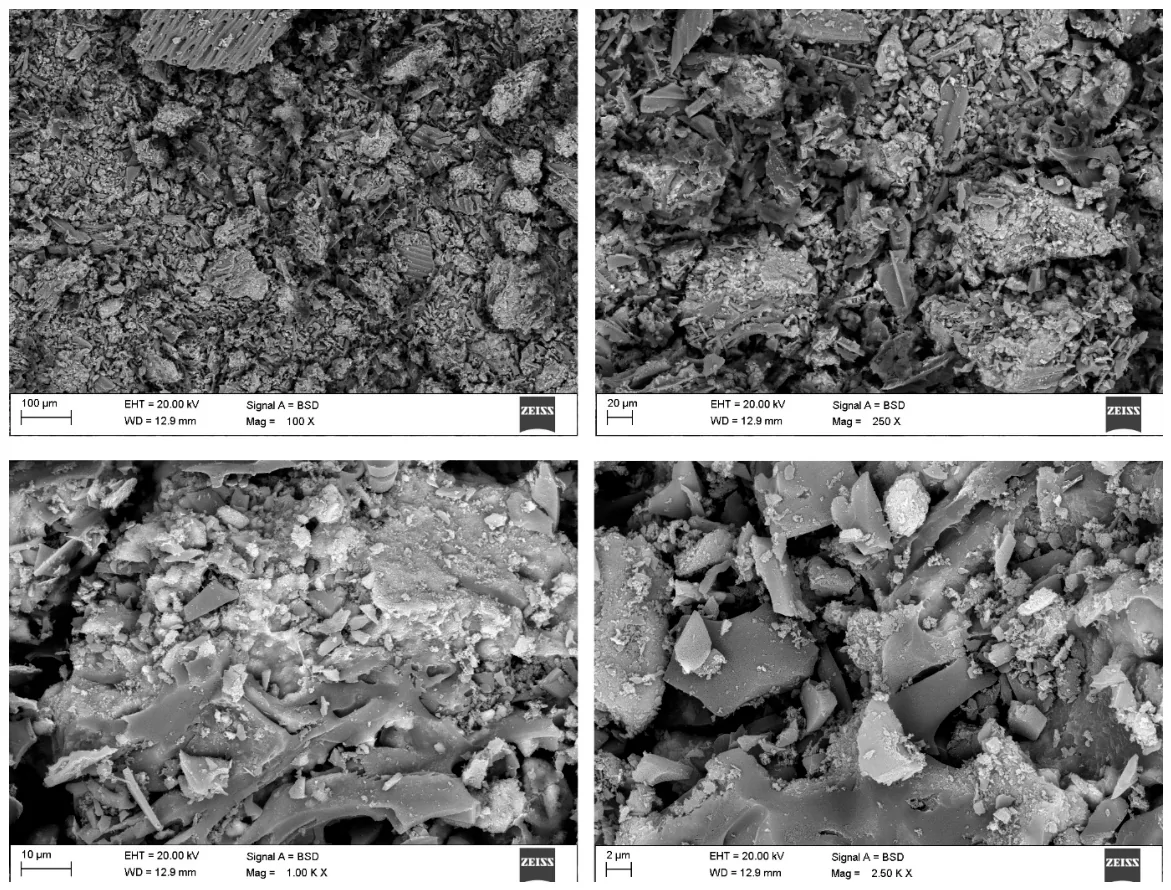
SEM images of studied mineral–carbon composite (D-C-HNT), magnification 100×, 250×, 1000×, 2500×.

The introduction of pyrolyzed pine sawdust alters the structure of the composites, as observed in the micrographs for K-C-HNT in Figure 6 and the corresponding D-C-HNT material shown in Figure 8. At lower magnifications of 100× and 250×, these composites are characterized by larger, blocky particles that retain the original structure of the pine wood. The SEM images highlight a highly porous structure featuring the macroporous, cellular microchannels typical of carbonized lignocellulosic biomass. Higher magnification images at 1000× and 2500× reveal that the HNT-binder mixture adheres to the surface and partially fills the macropores of the pyrolyzed sawdust. Moreover, the dense packing and encapsulation of HNTs within the carbonized binders (casein and dextrin) mirror the morphological observations reported for other composites, where the carbonaceous matrix integrates with the mineral particles [39].

**Figure 9:**
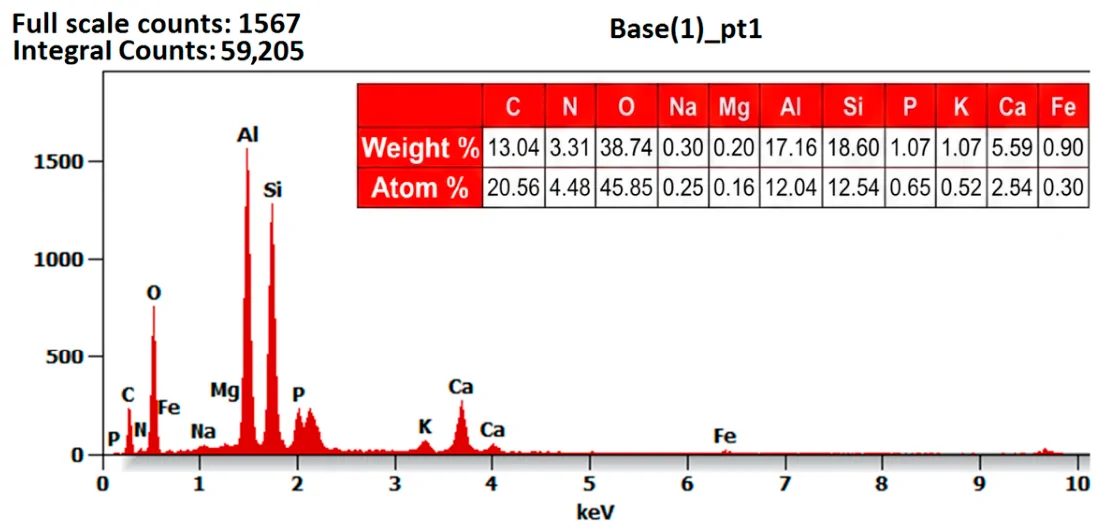
EDS characterization of the K-HNT composite.

**Figure 10:**
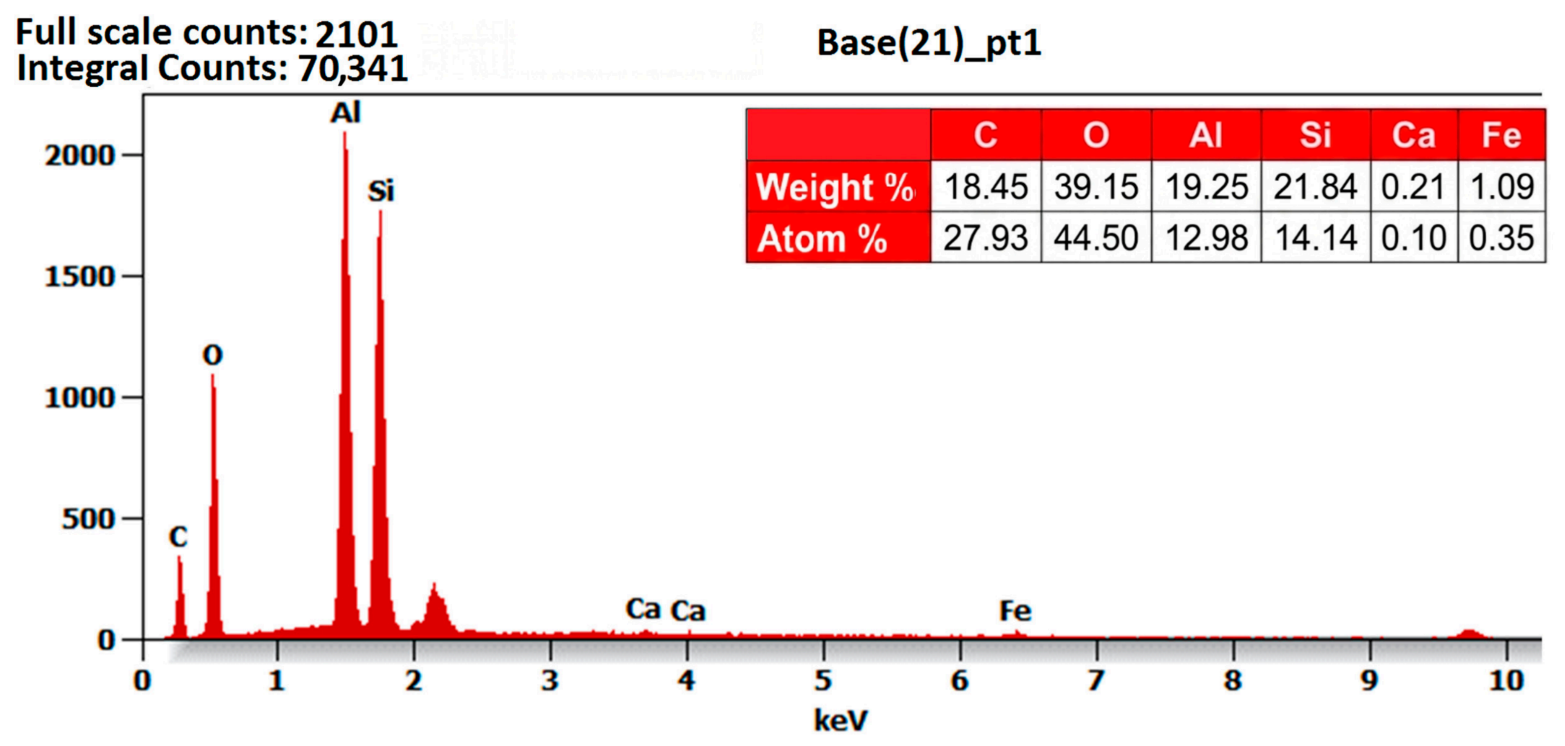
EDS characterization of the D-HNT composite.

Energy dispersive spectroscopy (EDS) was performed to determine the elemental composition of the synthesized mineral–carbon composites. The EDS spectrum of the K-HNT composite (Figure 9) confirms the presence of carbon (13.04 wt. %), oxygen (38.74 wt. %), aluminum (17.16 wt. %), and silicon (18.60 wt. %) as the primary elements. Minor amounts of nitrogen, sodium, magnesium, phosphorus, potassium, calcium, and iron were also detected in the K-HNT sample. Similar trends are observed for the dextrin-based material. The D-HNT composite (Figure 10) is primarily composed of carbon (18.45 wt. %), oxygen (39.15 wt. %), aluminum (19.25 wt. %), silicon (21.84 wt. %), and minor inclusions of calcium and iron. The prominent presence of aluminum and silicon in EDS spectra originates from the halloysite nanotubes (HNTs), confirming the incorporation of this mineral into the synthesized composites.

**Figure 11:**
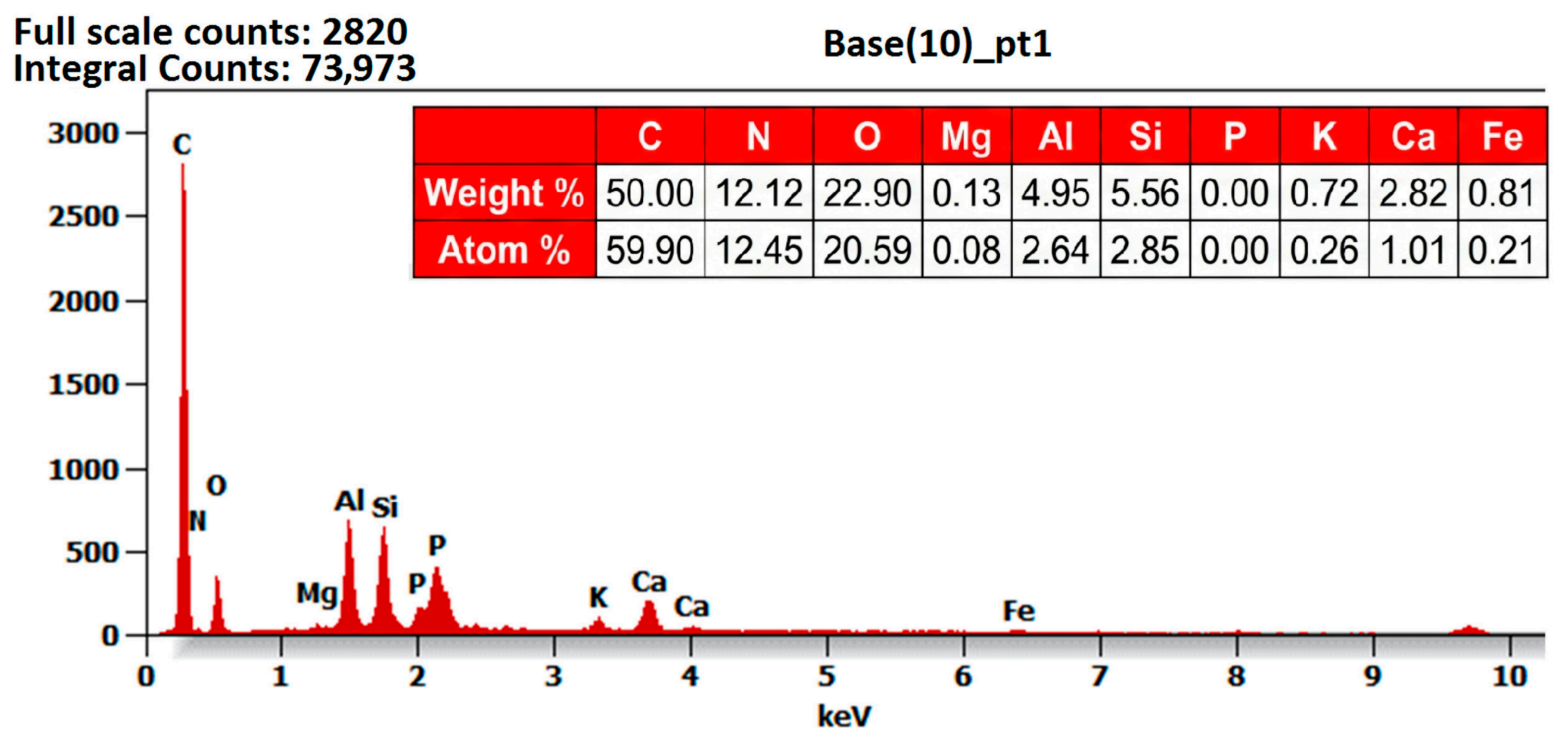
EDS characterization of the K-C-HNT composite.

**Figure 12:**
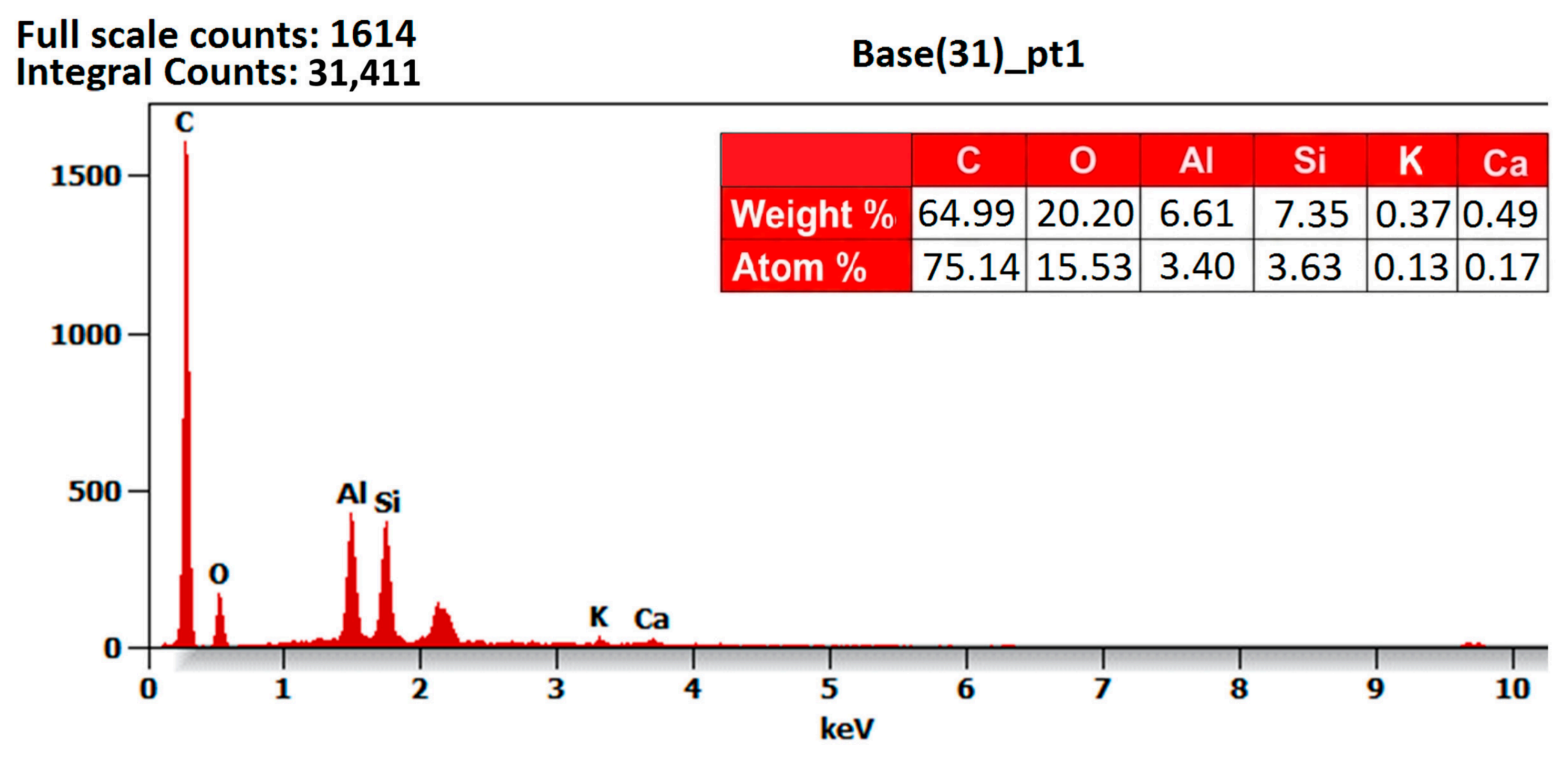
EDS characterization of the D-C-HNT composite.

The EDS analysis of the K-C-HNT composite (Figure 11) reveals its elemental composition. This material is predominantly composed of carbon from the addition of pyrolyzed pine sawdust, which accounts for 50.00 wt. % and oxygen at 22.90 wt. %. The presence of the halloysite nanotubes within the structure is confirmed by the detection of aluminum (4.95 wt. %) and silicon (5.56 wt. %). Additionally, the composite contains a notable amount of nitrogen at 12.12 wt. %, along with minor quantities of calcium (2.82 wt. %), iron (0.81 wt. %), potassium (0.72 wt. %), and magnesium (0.13 wt. %). Similarly, the EDS spectra of the D-C-HNT composite (Figure 12) demonstrates a highly carbonaceous structure with carbon constituting 64.99 wt. % of the material. Oxygen is the second most abundant element, present at 20.20 wt. %. The structural elements of the halloysite nanotubes are again visible, with silicon and aluminum accounting for 7.35 wt. % and 6.61 wt. %, respectively. Minor trace elements include calcium (0.49 wt. %) and potassium (0.37 wt. %).

### 2.5. The Effect of Eluent on SPE Efficiency

In this study, the effect of three eluents, acetone, ethyl acetate-n-hexane (1:1, *v*/*v*), and dichloromethane, on the efficiency of the SPE procedure was investigated. Each composite was evaluated for six organophosphorus pesticides (chlorpyrifos, disulfoton, ethoprophos, fenchlorphos, parathion methyl, and prothiofos). Azinphos methyl and dichlorvos were excluded from the SPE procedure due to their weak analytical signal during GC-MS/MS analysis.

**Figure 13:**
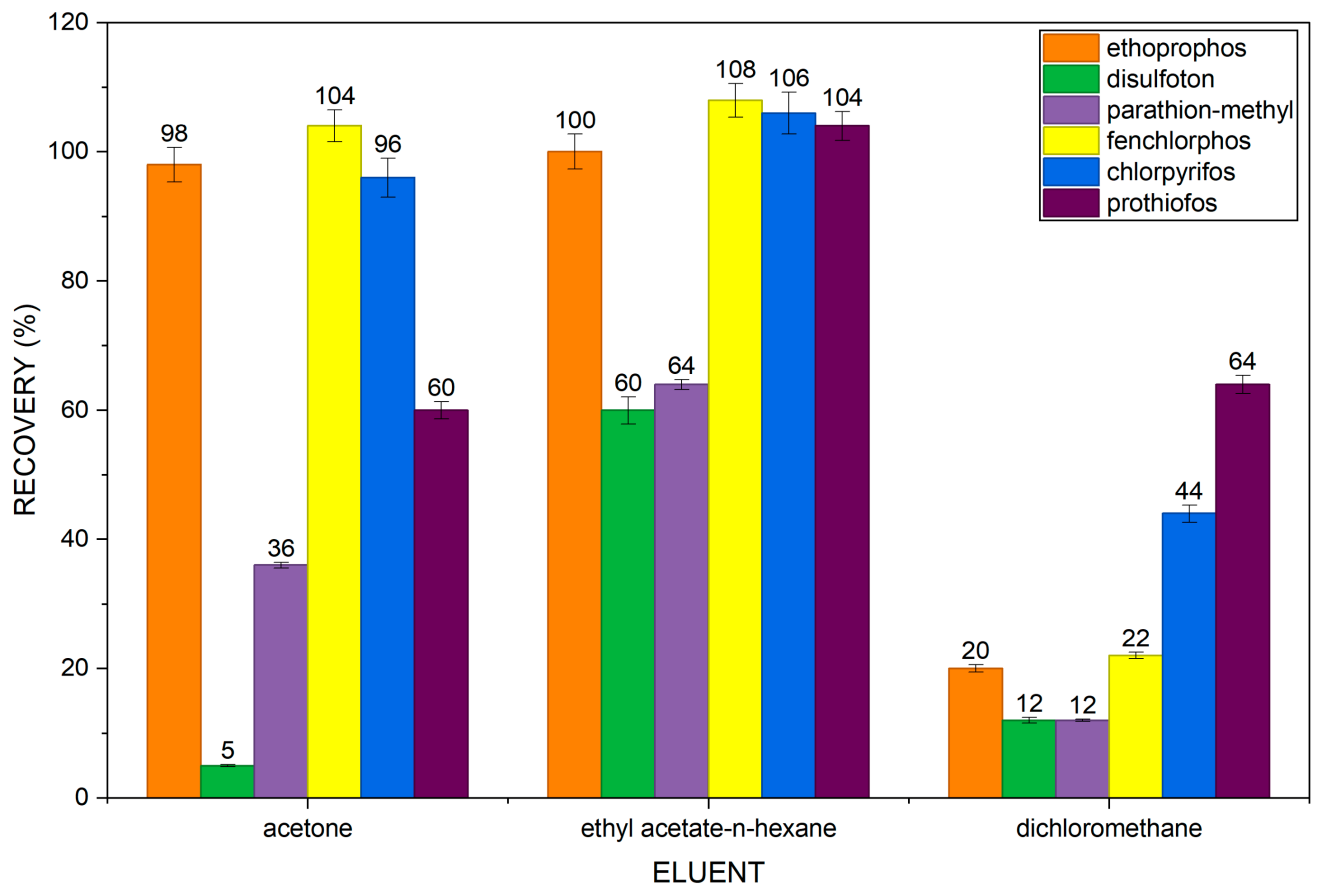
Effect of the eluent on SPE efficiency for 50 mg of K-HNT composite, 10 cm^3^ of sample loading volume, initial concentration of each pesticide: 50 μg dm^−3^.

**Figure 14:**
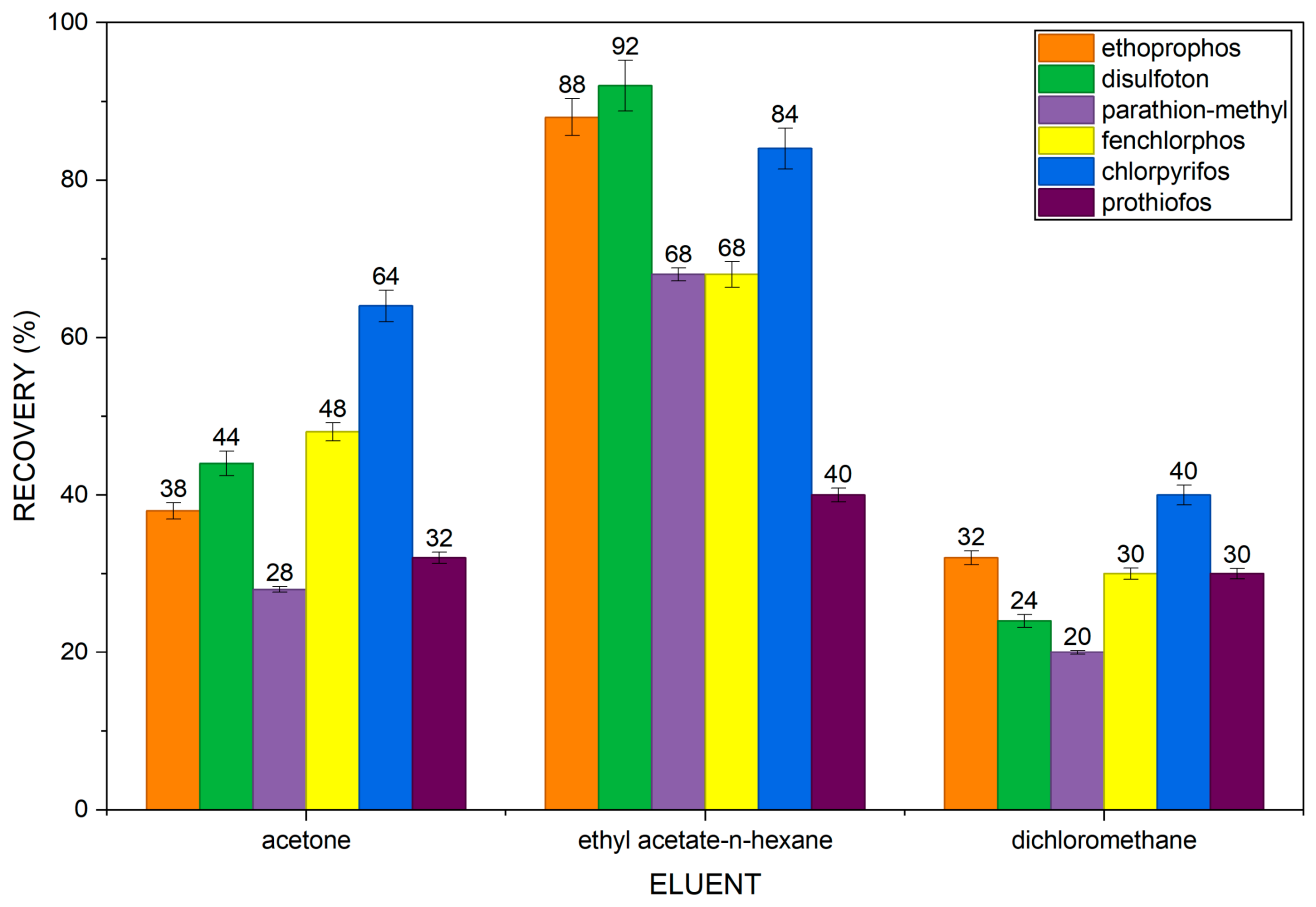
Effect of the eluent on SPE efficiency for 50 mg of K-C-HNT composite, 10 cm^3^ of sample loading volume, initial concentration of each pesticide: 50 μg dm^−3^.

**Figure 15:**
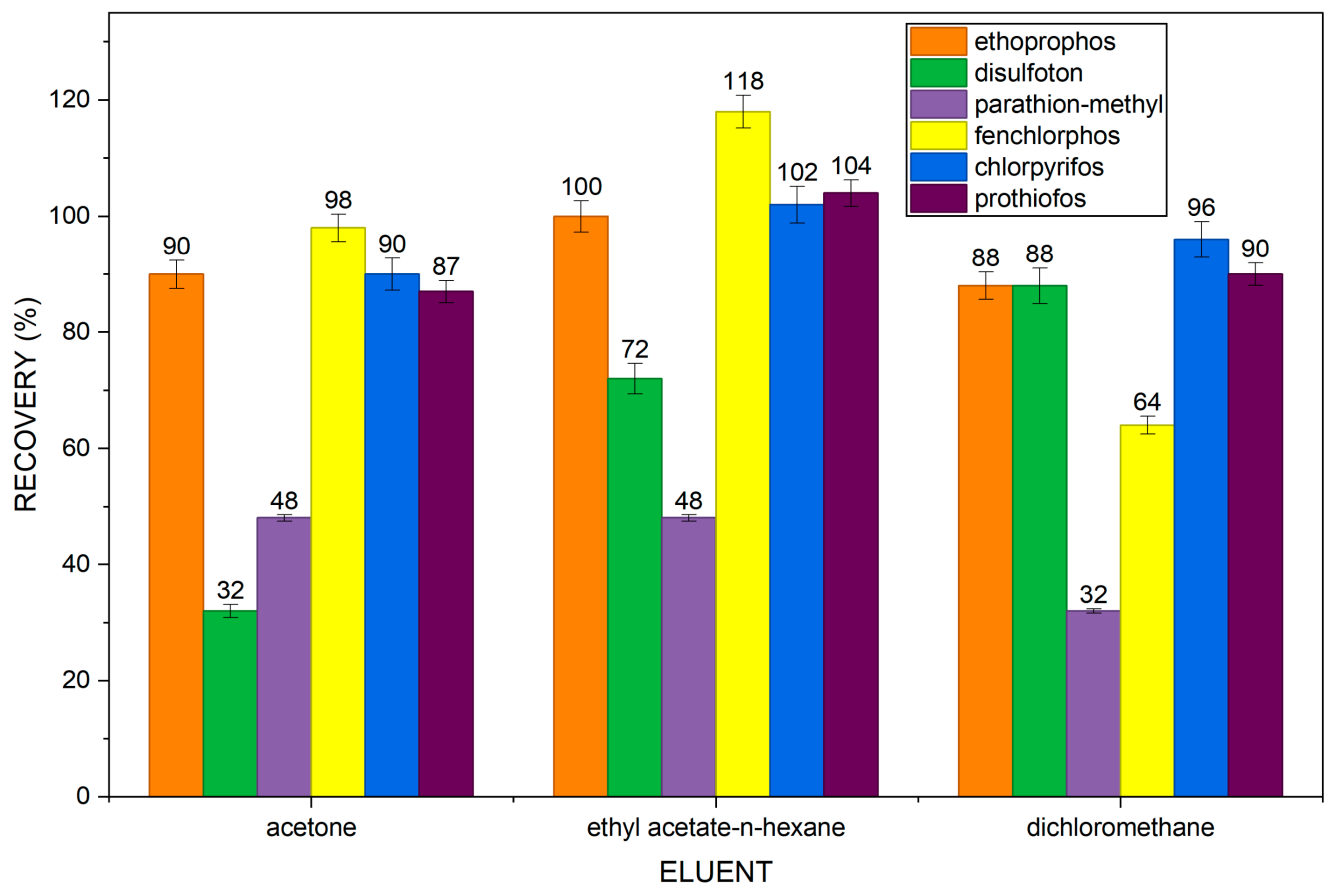
Effect of the eluent on SPE efficiency for 50 mg of D-HNT composite, 10 cm^3^ of sample loading volume, initial concentration of each pesticide: 50 μg dm^−3^.

**Figure 16:**
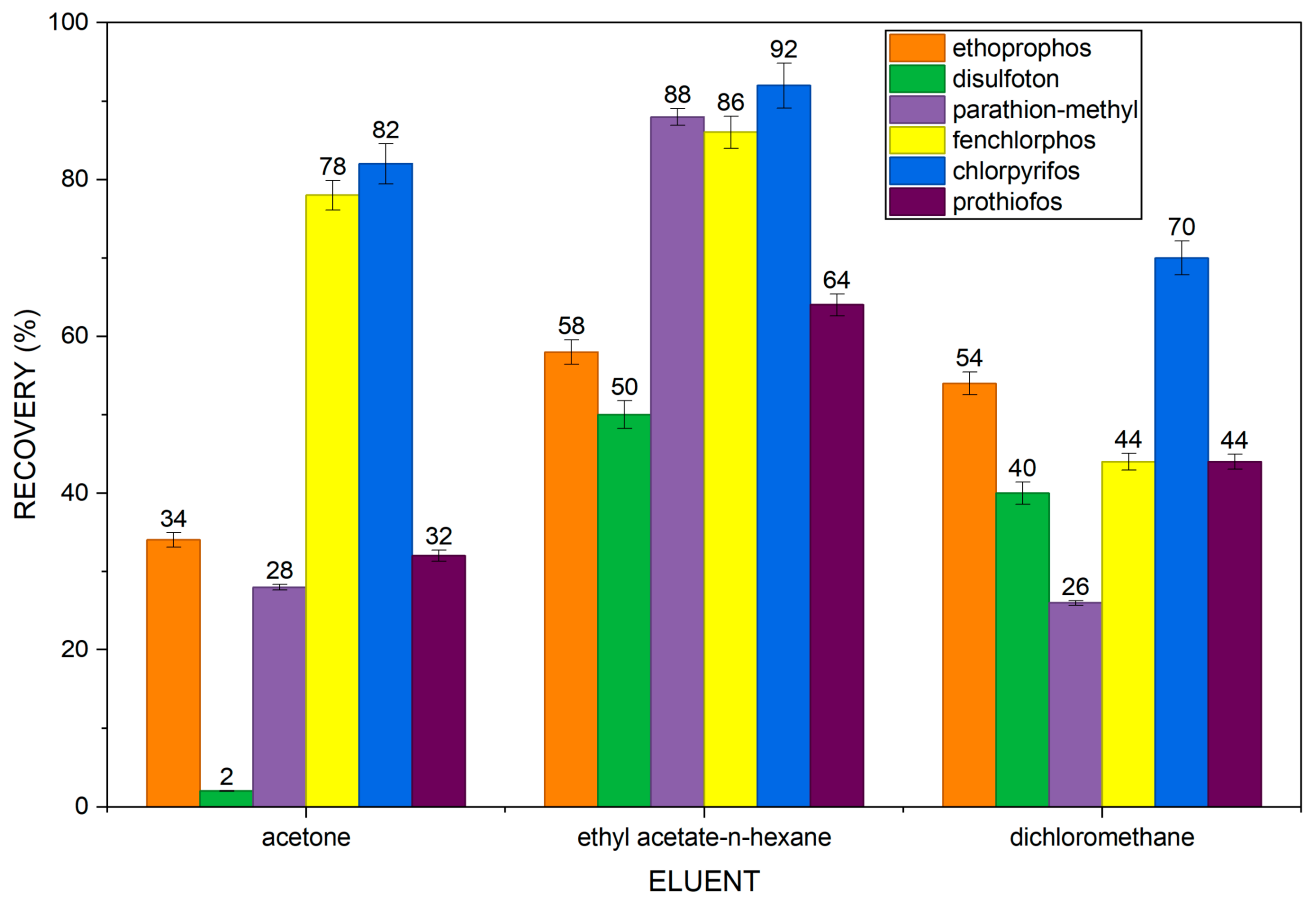
Effect of the eluent on SPE efficiency for 50 mg of D-C-HNT composite, 10 cm^3^ of sample loading volume, initial concentration of each pesticide: 50 μg dm^−3^.

The studies were conducted for all four materials, with the results for the casein-based composites (K-HNT and K-C-HNT) presented in Figure 13 and Figure 14 and the results for the dextrin-based sorbents (D-HNT and D-C-HNT) shown in Figure 15 and Figure 16. The most effective eluent should be characterized by high elution of all six pesticides used in the experiment. Based on the obtained results, it was concluded that the best eluent for all obtained mineral–carbon composites was an ethyl acetate-n-hexane solution (1:1, *v*/*v*).

Figure 13 presents the results for the K-HNT material. For the ethyl acetate-n-hexane solution, the highest recoveries ranging from 100 to 108% were obtained for the following pesticides: ethoprophos, fenchlorphos, chlorpyrifos, and prothiofos. For acetone, values ranging from 96 to 104% were obtained for ethoprophos, fenchlorphos, and chlorpyrifos. In contrast, the recoveries obtained with dichloromethane were unsatisfactory, remaining below 64%.

Figure 14 presents the recovery values for the K-C-HNT mineral–carbon composite. Once again, the ethyl acetate-n-hexane (1:1, *v*/*v*) mixture provided the highest recovery values ranging from 84% to 92% for three pesticides: ethoprophos, disulfoton, and chlorpyrifos. The results for acetone were below 64% and for dichloromethane below 40% for all examined pesticides.

Figure 15 presents the influence of the eluent on the SPE efficiency of the dextrin-based material (D-HNT). When using the ethyl acetate-n-hexane mixture, the recoveries of ethoprophos, fenchlorphos, chlorpyrifos, and prothiofos ranged from 100% to 118%. For acetone, the recovery values for these four pesticides were slightly lower but still satisfactory, falling within the range of 87–98%. In the case of dichloromethane, the recoveries were higher compared to those obtained for the casein-based materials and ranged from 88% to 96% for ethoprophos, disulfoton, chlorpyrifos, and prothiofos.

In Figure 16, recovery values for the D-C-HNT mineral–carbon composite are shown. Using the ethyl acetate-n-hexane mixture, the highest recoveries were observed for parathion-methyl, fenchlorphos, and chlorpyrifos, ranging from 86% to 92%. In the case of acetone, only two pesticides, fenchlorphos (78%) and chlorpyrifos (82%), exhibited satisfactory recoveries, while the remaining analytes showed markedly lower values. For dichloromethane, the highest recovery was obtained for chlorpyrifos (70%), whereas recoveries for all other pesticides were lower.

It should be noted that because solid-phase extraction (SPE) is a dynamic, flow-through analytical method operating under non-equilibrium conditions, quantitative recovery does not prove that thermodynamic equilibrium was reached.

Because SPE is a dynamic process, static adsorption mechanisms cannot be validated without independent kinetic studies. However, the high extraction efficiency can be explained by plausible analyte–sorbent interactions based on the composites’ physical properties. The porous structure (confirmed by BET and SEM) reduces mass-transfer resistance and allows rapid analyte diffusion during sample loading. Meanwhile, the turbostratic carbon coatings (confirmed by Raman and FT-IR) cover the HNT surface, providing essential hydrophobic active sites and aromatic domains. These structural features facilitate plausible analyte–sorbent interactions that are highly dependent on the chemical structure of the specific pesticide. For aromatic analytes (parathion-methyl, fenchlorphos, chlorpyrifos, and prothiofos), the extraction is facilitated by a combination of hydrophobic interactions and plausible π–π stacking with the conjugated carbonaceous domains. In contrast, for non-aromatic, aliphatic compounds like ethoprophos and disulfoton, the extraction is driven exclusively by hydrophobic (van der Waals) interactions between their alkyl chains and the non-polar surfaces of the composites. This differentiated behavior is consistent with the retention pathways widely reported for other carbon-based sorbents [20,34,35]. Furthermore, because the SPE procedure was conducted using a multi-component pesticide mixture to simulate real environmental conditions, the observed analyte-dependent recovery trends are also influenced by simultaneous, competitive co-adsorption at the heterogeneous active sites.

The observed trends indicate that the extraction performance of the mineral–carbon composites cannot be explained by the BET surface area alone. For instance, although the D-C-HNT composite possesses the highest specific surface area (218.3 m^2^ g^−1^) and the highest carbon content (64.99 wt. %), it did not consistently exhibit the highest recoveries. This behavior is related to its pore-size distribution, which is predominantly microporous (only 36.06% mesoporosity). Organophosphorus pesticides are relatively large, bulky molecules that experience significant steric hindrance when diffusing into narrow micropores, especially under the short contact times characteristic of SPE flow. In contrast, composites such as K-HNT and D-HNT, despite having lower overall surface areas (44.2 and 115.6 m^2^ g^−1^, respectively) and lower carbon contents, produced higher extraction efficiencies. This is due to their highly developed mesoporous architectures (93.21% and 61.48% mesoporosity, respectively). The abundance of mesopores provides unhindered mass-transfer channels, allowing the large analyte molecules to access the hydrophobic and aromatic active sites on the carbonized surface.

Thus, the synergistic combination of accessible mesoporosity and sufficient carbon coating is the primary driver of SPE performance for these bulky analytes, rather than absolute surface area. As demonstrated in recent studies, a systematic evaluation of structure and properties is crucial to fully understand how complex, mixed-phase materials perform [40].

### 2.6. The Effect of the Mineral–Carbon Composite Mass on the SPE Efficiency

**Figure 17:**
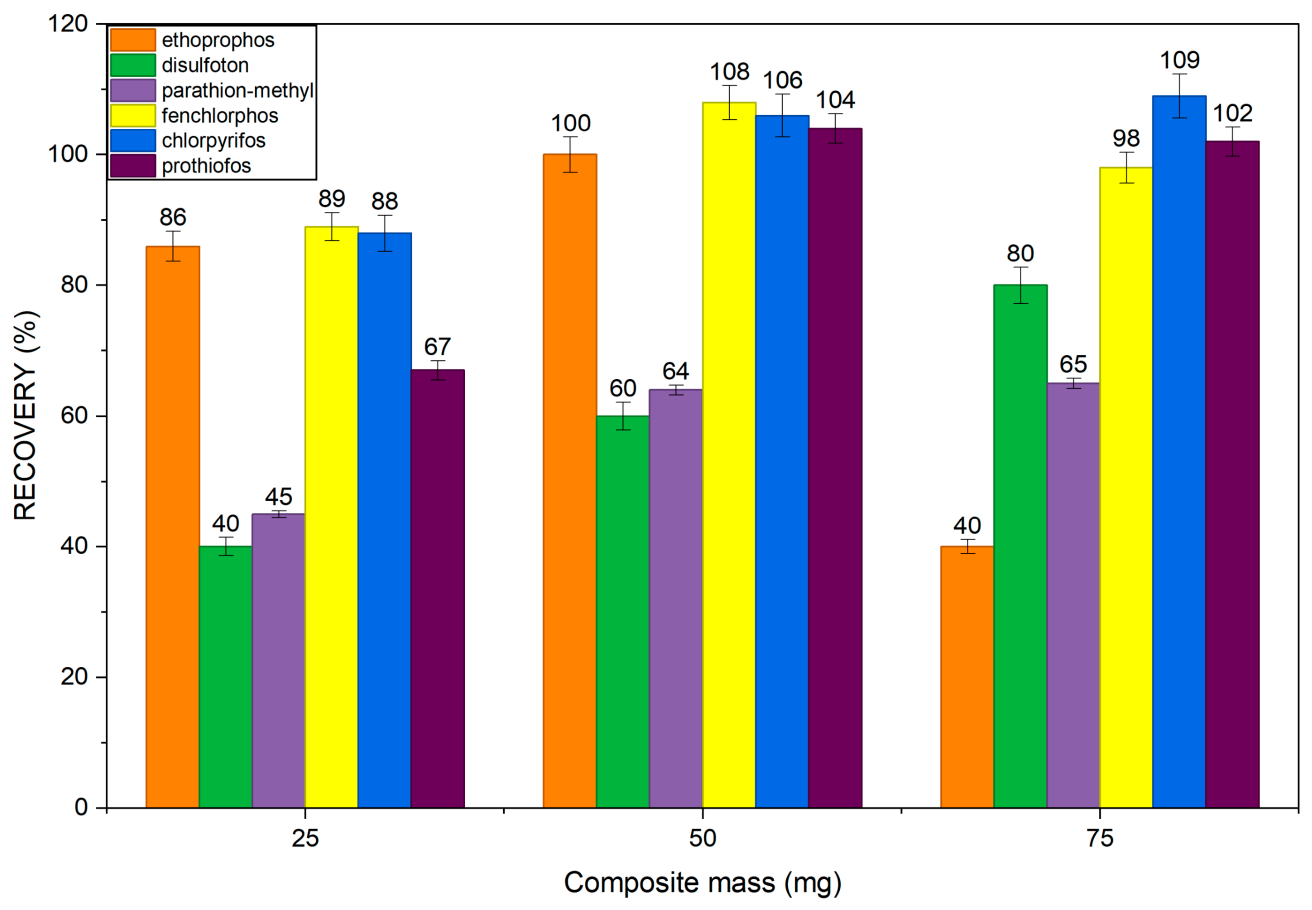
Effect of the K-HNT composite mass on SPE efficiency using ethyl acetate-n-hexane (1:1, *v*/*v*) as the eluent, 10 cm^3^ of sample loading volume, initial concentration of each pesticide: 50 μg dm^−3^.

**Figure 18:**
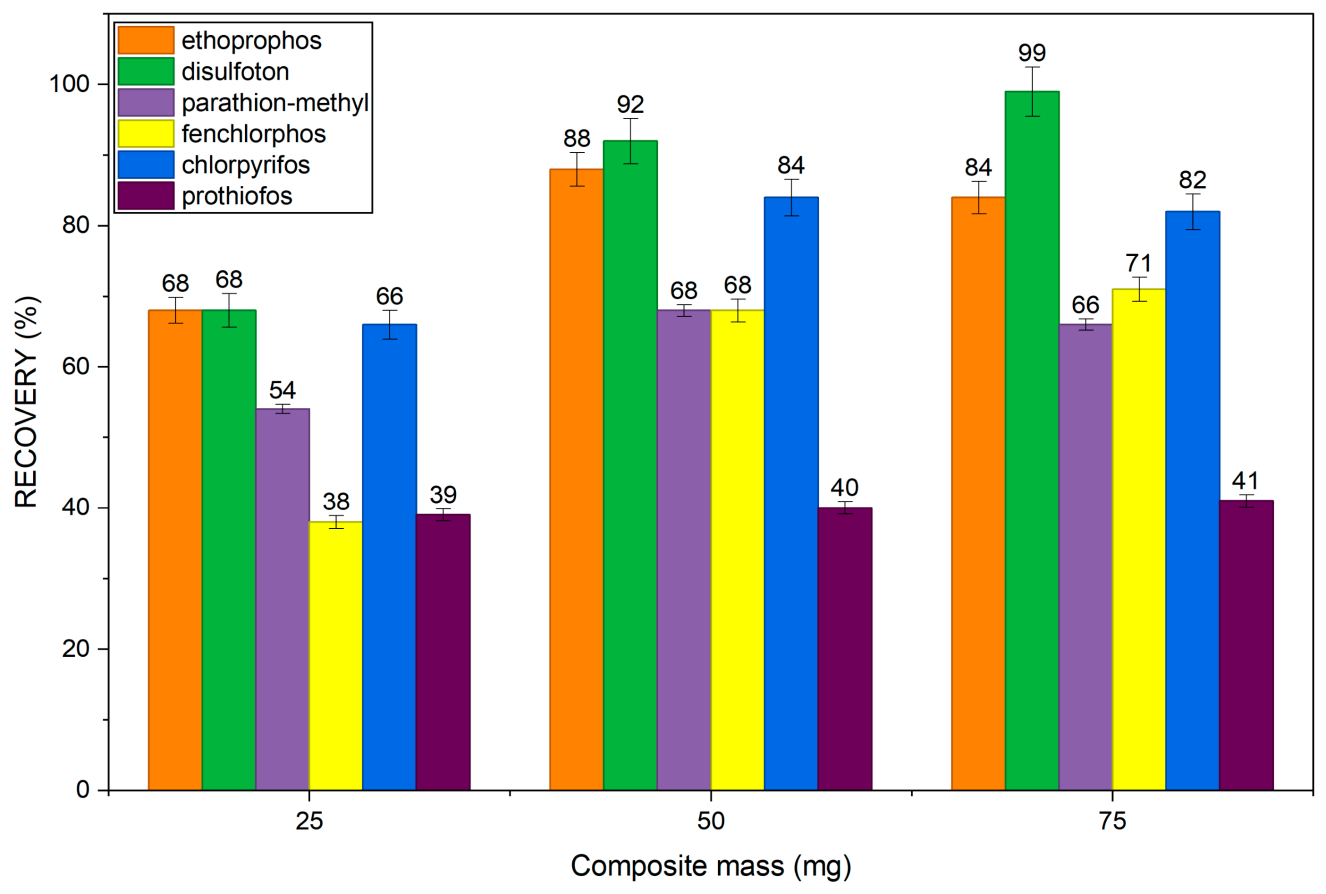
Effect of the K-C-HNT composite mass on SPE efficiency using ethyl acetate-n-hexane (1:1, *v*/*v*) as the eluent, 10 cm^3^ of sample loading volume, initial concentration of each pesticide: 50 μg dm^−3^.

**Figure 19:**
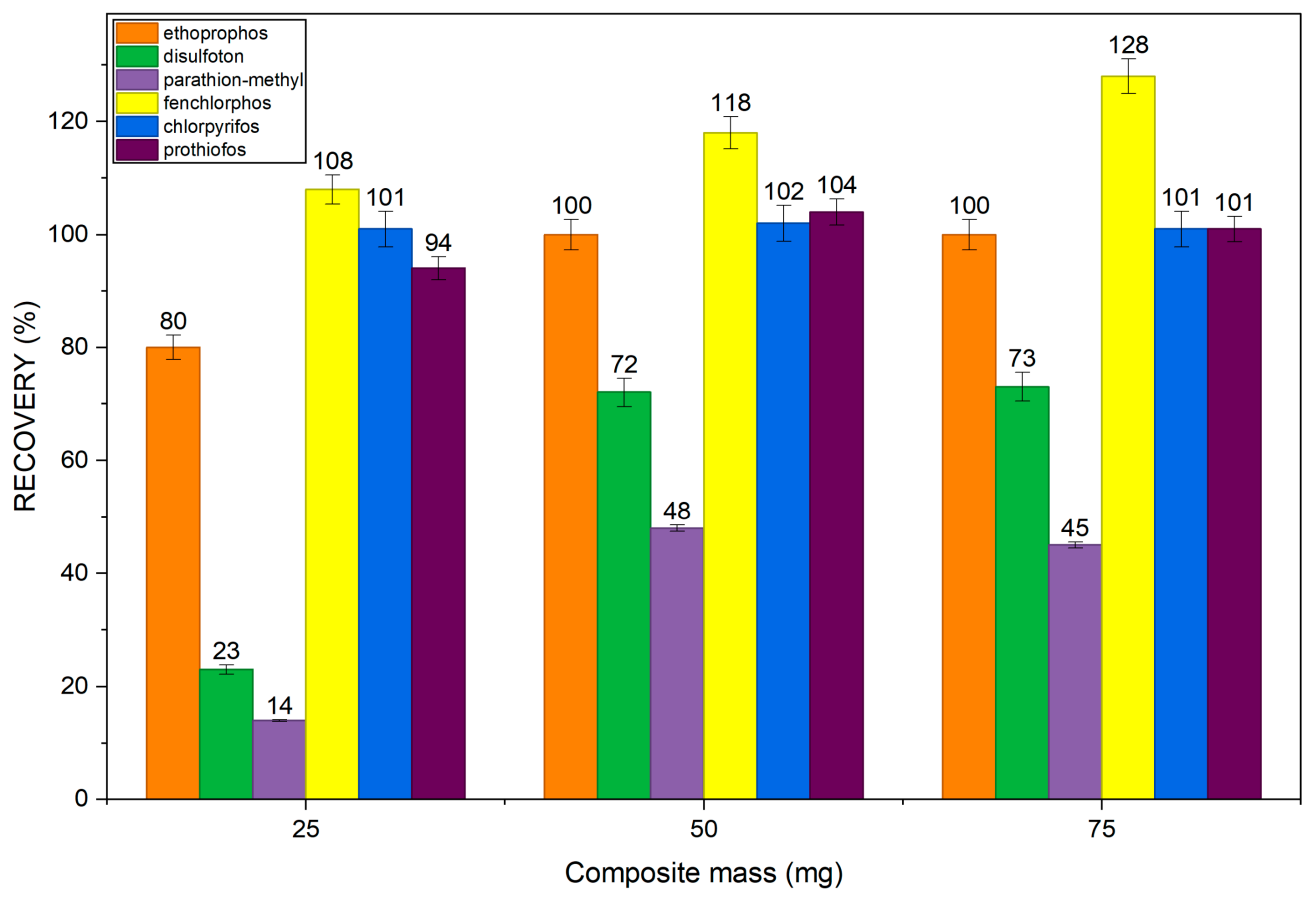
Effect of the D-HNT composite mass on SPE efficiency using ethyl acetate-n-hexane (1:1, *v*/*v*) as the eluent, 10 cm^3^ of sample loading volume, initial concentration of each pesticide: 50 μg dm^−3^.

**Figure 20:**
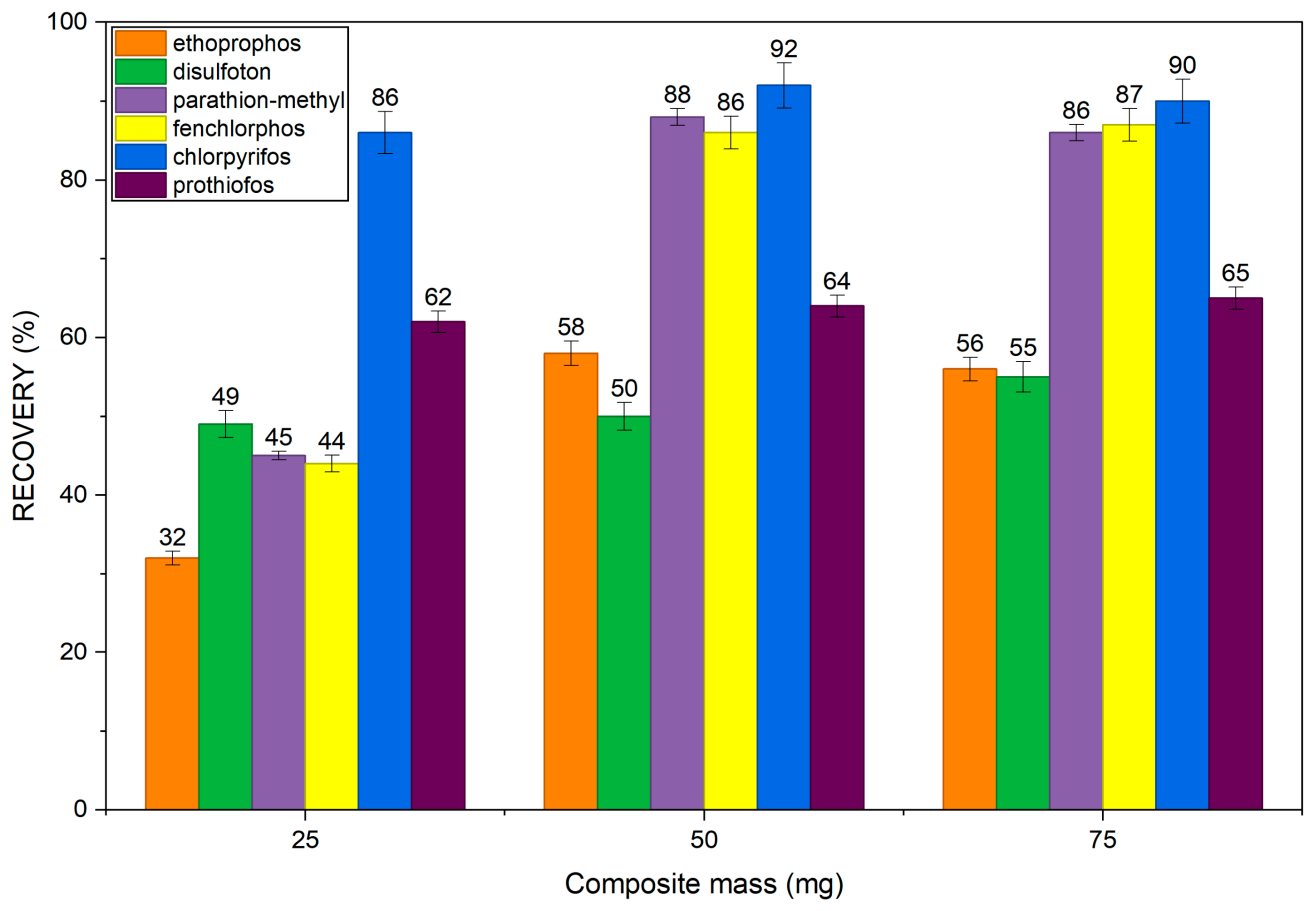
Effect of the D-C-HNT composite mass on SPE efficiency using ethyl acetate-n-hexane (1:1, *v*/*v*) as the eluent, 10 cm^3^ of sample loading volume, initial concentration of each pesticide: 50 μg dm^−3^.

To evaluate the effect of sorbent mass on SPE performance, the SPE cartridges were loaded with all tested adsorbents, including K-HNT, K-C-HNT, D-HNT, and D-C-HNT. The results are presented in Figure 17, Figure 18, Figure 19 and Figure 20, respectively. Sorbent masses of 25, 50, and 75 mg were tested, and an ethyl acetate-n-hexane solution (1:1, *v*/*v*) was used as the eluent, as it achieved the highest recovery values for the examined pesticides. Figure 17 presents the results for the K-HNT composite. For a sorbent mass of 25 mg, the recovery values were 86% for ethoprophos, 89% for fenchlorphos, and 88% for chlorpyrifos. At 50 mg, the values were higher and ranged from 100 to 108% for four pesticides: ethoprophos, fenchlorphos, chlorpyrifos, and prothiofos. At 75 mg, values ranging from 98 to 109% were obtained for three pesticides, including fenchlorphos, chlorpyrifos, and prothiofos.

Figure 18 presents the results obtained for the K-C-HNT material. In this case, a sorbent mass of 25 mg was inadequate for the SPE procedure, with recovery values below 68%. Increasing the mass to 50 mg resulted in noticeably higher recoveries, particularly for ethoprophos, disulfoton, and chlorpyrifos, which ranged from 84% to 92%. At 75 mg, the recovery values were comparable across all analyzed pesticides, with the highest recoveries again observed for ethoprophos, disulfoton, and chlorpyrifos, ranging from 84% to 99%.

Figure 19 shows the results obtained for the dextrin-based material (D-HNT). For a sorbent mass of 25 mg, recovery values ranging from 80% to 108% were recorded for four pesticides: ethoprophos, fenchlorphos, chlorpyrifos, and prothiofos. These same pesticides also demonstrated the highest recoveries at 50 mg, reaching 100–118%, as well as at 75 mg, where recoveries increased further to 100–128%. Among all tested sorbent masses, fenchlorphos consistently achieved the highest recovery value.

The last studied mineral–carbon composite was D-C-HNT, as shown in Figure 20. For a sorbent mass of 25 mg, only chlorpyrifos exhibited a relatively high recovery of 86%, while the remaining pesticides were considerably lower (32–62%). At 50 mg, parathion-methyl, fenchlorphos, and chlorpyrifos showed improved recoveries, ranging from 88% to 92%. For a sorbent mass of 75 mg, values were comparable to those obtained at 50 mg for all tested pesticides. It should be noted that in the context of analytical SPE for trace analysis, the mass of the analytes is at the microgram level; thus, the sorbent bed operates well below its maximum adsorption capacity, preventing column overload and ensuring quantitative recoveries.

It should be noted that the majority of extraction recoveries fall within the generally accepted optimal analytical range of 70–120% [41]. While positive analytical biases slightly exceeding 100% are a known phenomenon in trace pesticide analysis [42], the occasional extreme values observed in our study (reaching up to 128% for the D-HNT sorbent at 75 mg) fall strictly outside optimal quantitative limits [41]. This positive bias is likely a cumulative result of several analytical factors, including the relatively modest calibration linearity for certain analytes (R^2^ = 0.96–0.98), potential minor volumetric deviations during the extraction and solvent evaporation steps, and unquantified co-extractives originating from the complex, waste-derived carbonaceous matrices. However, while recoveries exceeding 120% fall outside standard limits [41], they remain analytically acceptable under specific conditions. According to the European Commission SANTE guidelines for pesticide residue analysis (SANTE/11312/2021 v2026), mean recoveries outside the 70–120% range (up to a maximum of 140%) can be accepted in exceptional cases, provided they demonstrate consistent repeatability with a relative standard deviation (RSD) ≤ 20% [43]. As demonstrated by the low variance across our replicates, the RSD for the 128% recovery meets this criterion. Thus, this result confirms the composites’ strong extraction affinity and remains within acceptable regulatory limits for complex matrices without internal standards. However, future regulatory water monitoring will require rigorous matrix-effect evaluations.

### 2.7. Multiple SPE Extraction

**Figure 21:**
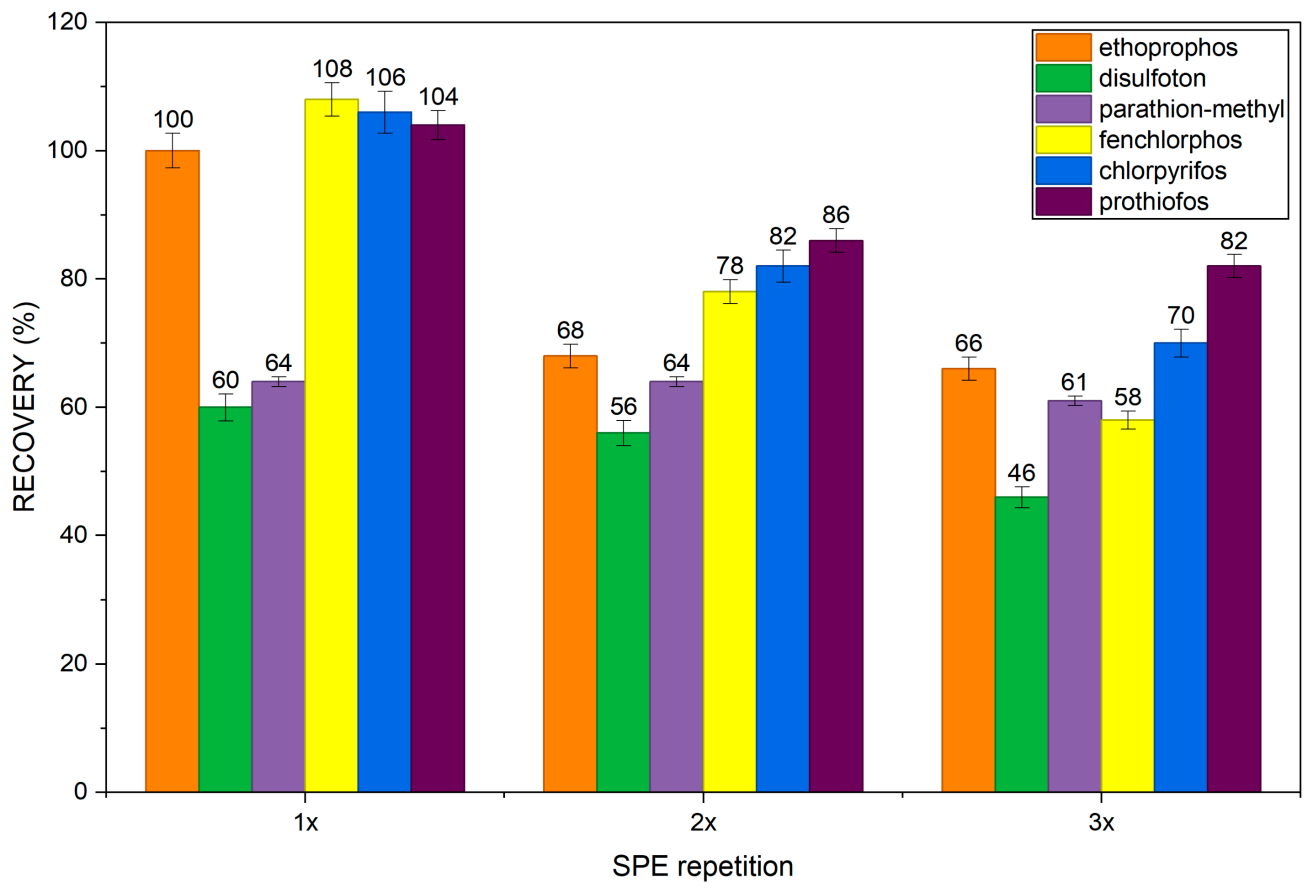
Effect of the multiple extraction on SPE efficiency for 50 mg of K-HNT composite, ethyl acetate-n-hexane (1:1, *v*/*v*) as the eluent, 10 cm^3^ of sample loading volume, initial concentration of each pesticide: 50 μg dm^−3^.

**Figure 22:**
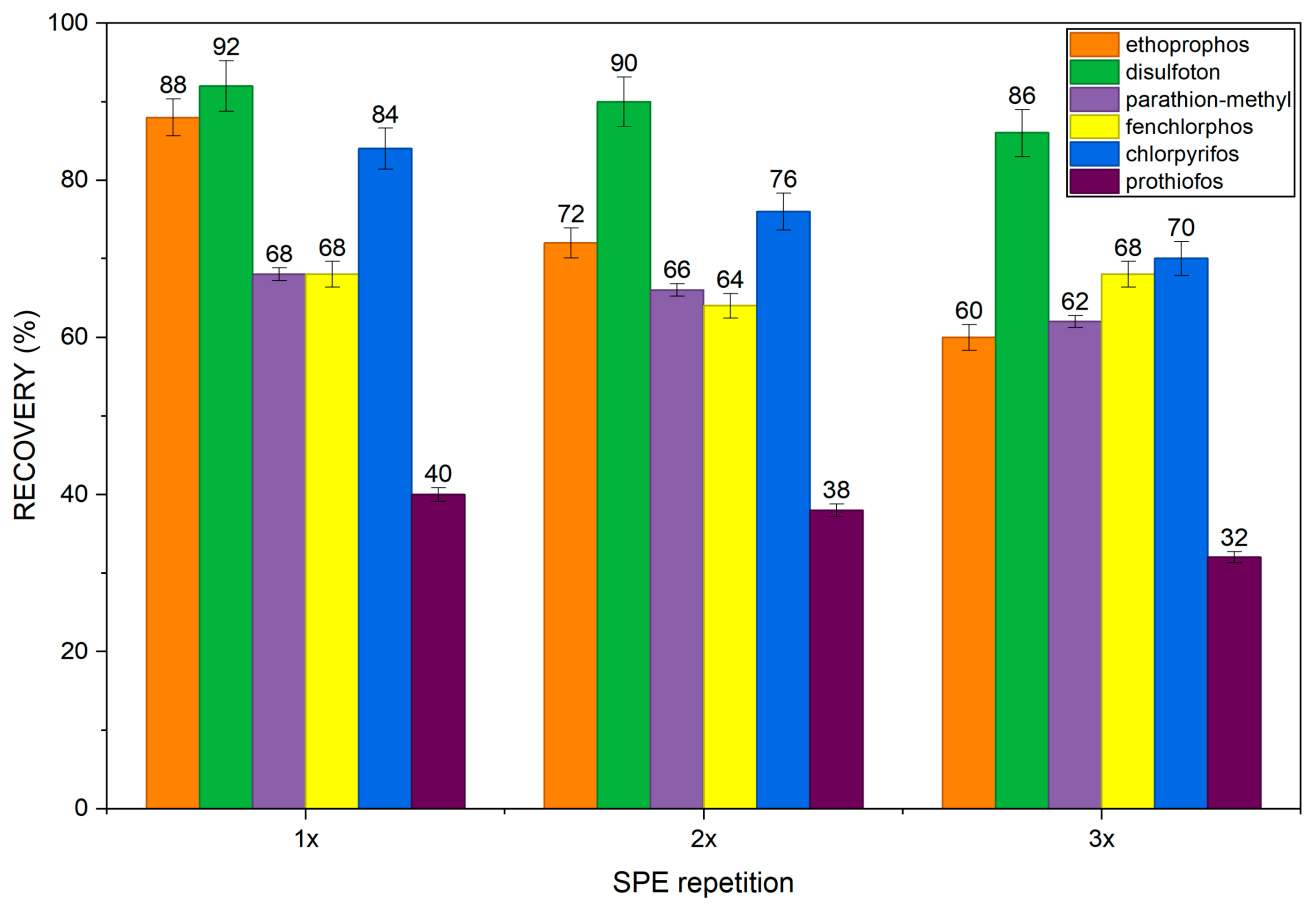
Effect of the multiple extraction on SPE efficiency for 50 mg of K-C-HNT composite, ethyl acetate-n-hexane (1:1, *v*/*v*) as the eluent, 10 cm^3^ of sample loading volume, initial concentration of each pesticide: 50 μg dm^−3^.

**Figure 23:**
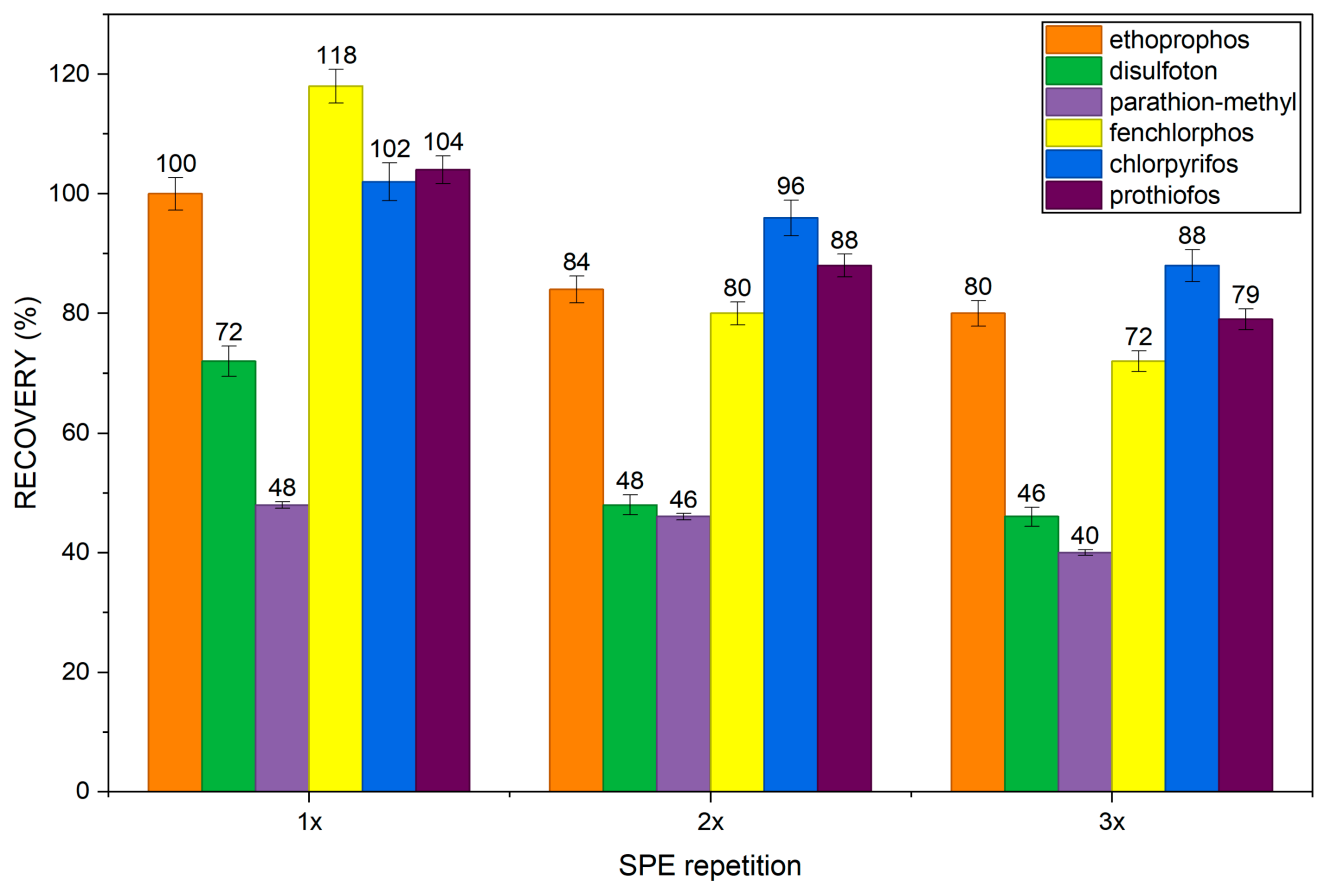
Effect of the multiple extraction on SPE efficiency for 50 mg of D-HNT composite, ethyl acetate-n-hexane (1:1, *v*/*v*) as the eluent, 10 cm^3^ of sample loading volume, initial concentration of each pesticide: 50 μg dm^−3^.

**Figure 24:**
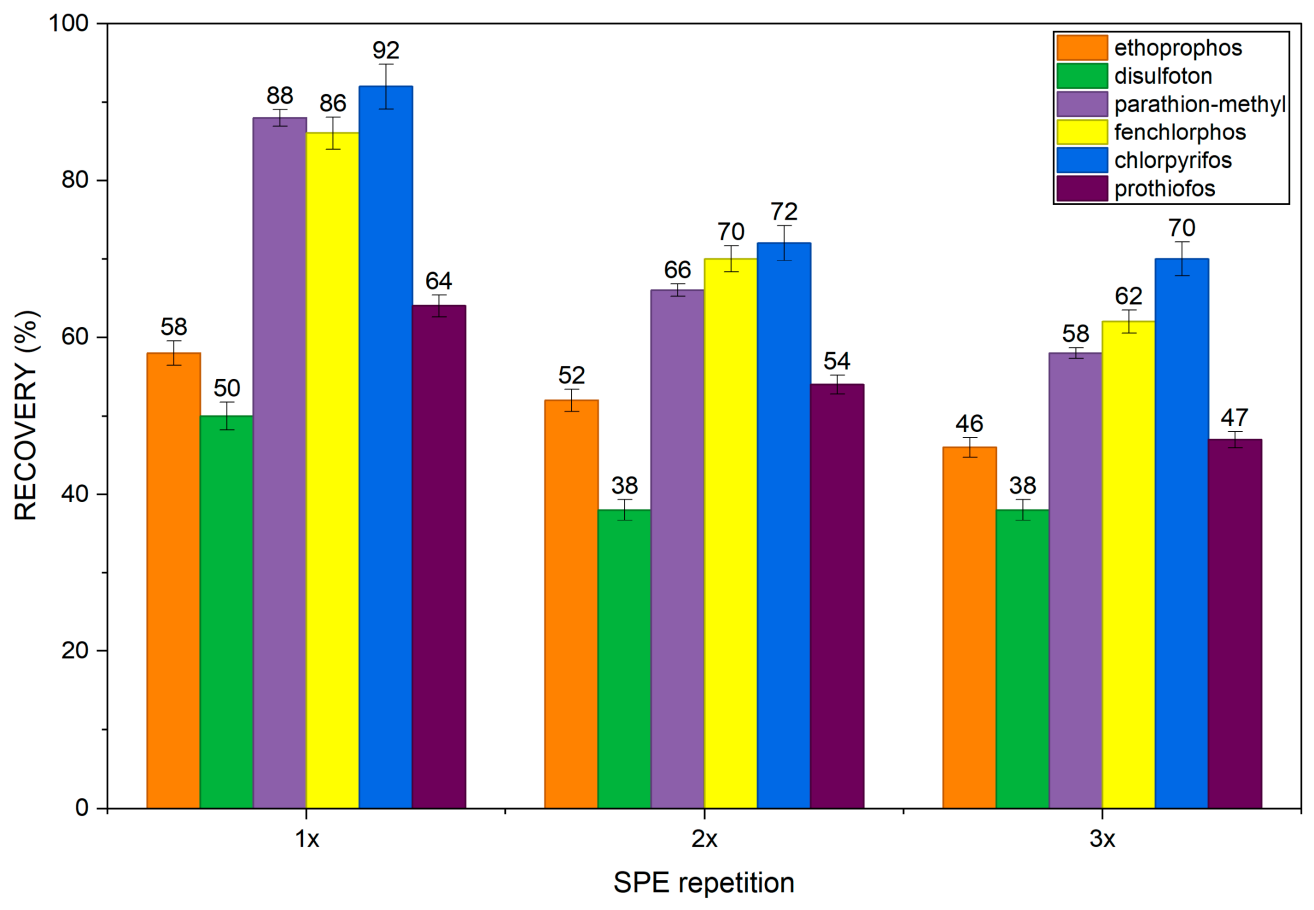
Effect of the multiple extraction on SPE efficiency for 50 mg of D-C-HNT composite, ethyl acetate-n-hexane (1:1, *v*/*v*) as the eluent, 10 cm^3^ of sample loading volume, initial concentration of each pesticide: 50 μg dm^−3^.

Multiple SPE extractions were performed for all tested materials (K-HNT, K-C-HNT, D-HNT, and D-C-HNT). Each SPE cartridge was packed with 50 mg of the selected sorbent. As the eluent, a mixture of ethyl acetate-n-hexane (1:1, *v*/*v*) was used. Two consecutive extractions were carried out without replacing the SPE cartridge packing. After the first extraction, the column was rinsed with 2 cm^3^ of methanol and placed in the laboratory dryer at 100 °C for 30 min to evaporate any residual solvent. Once dried, the SPE procedure was repeated following the method described in Section 2.5. The results obtained are presented in Figure 21, Figure 22, Figure 23 and Figure 24. The reusability was evaluated over 3 consecutive cycles to confirm the structural stability of the composites under the tested regeneration conditions.

Figure 21 shows the effect of repeated SPE procedure for the K-HNT material. In the first extraction, recovery values of 100–108% were observed for ethoprophos, fenchlorphos, chlorpyrifos, and prothiofos. In the second repetition, the recoveries decreased to 56–86% for all tested pesticides. The third time resulted in a slight decrease to 46–82%, although these values were comparable to those obtained in the second extraction.

Figure 22 shows the results for K-C-HNT. The highest results were observed for three pesticides: ethoprophos, disulfoton, and chlorpyrifos (84–92%). The second repetition reduced these values to 72–90%, and the third to 60–86%. For the remaining pesticides, the recovery values decreased slightly compared to the first SPE procedure.

Figure 23 shows the dextrin-based material D-HNT. The first SPE procedure given results of 100–118% for the four pesticides ethoprophos, fenchlorphos, chlorpyrifos, and prothiofos. The second repetition reduced these values to 80–96%, and the third to 72–88%.

Figure 24 presents the results for the D-C-HNT material. The highest recoveries (86–92%) were observed for the three pesticides parathion-methyl, fenchlorphos, and chlorpyrifos. These values decreased to 66–72% in the second extraction and to 58–70% in the third extraction. The remaining results were unsatisfactory for the SPE procedure. Furthermore, the results obtained in the second and third repetitions for all pesticides were comparable.

It must be emphasized that the observed decrease in recovery during SPE cycles is not associated with the saturation of the sorbents. The total mass of analytes loaded onto the column (e.g., ~1.5 µg over three cycles) is negligible compared to the 50 mg sorbent bed, meaning the materials operate far below their maximum adsorption capacities. Instead, the gradual reduction in extraction efficiency is likely attributed to the alteration of the sorbent surface properties induced by the elution solvents, thermal stress during the drying steps at 100 °C, and potential mechanical deformation of the sorbent bed caused by repeated vacuum applications.

### 2.8. Comparison of Extraction Efficiency with Pure Materials and Other Reported Sorbents

The synthesized mineral–carbon composites (K-HNT, D-HNT, K-C-HNT, and D-C-HNT) were compared with the pure precursor materials. For this reason, the carbon part consisting of pyrolyzed casein, dextrin, and pine sawdust, as well as the mineral phase, halloysite nanotubes (HNTs), were analyzed under identical analytical conditions. Results are shown in Table 3 for acetone and Table 4 for ethyl acetate-n-hexane (1:1, *v*/*v*) as the eluents. The data indicate that carbon precursors and the mineral phase exhibited lower recovery efficiencies in comparison to the composites. As for the acetone as the eluent, only dextrin exceeded a 70% recovery threshold, reaching 72% for disulfoton. With the ethyl acetate-n-hexane mixture, peak recoveries among the raw materials were observed for casein (72% ethoprophos), dextrin (70% chlorpyrifos), and pyrolyzed sawdust (74% prothiofos). Moreover, the halloysite nanotubes (HNTs) consistently yielded the lowest extraction efficiencies.

The results were also compared with well-known commercial sorbents, such as Florisil, active carbon, and C-18. These data are presented in Table 5 for acetone and Table 6 for ethyl acetate-n-hexane mixture (1:1, *v*/*v*) as eluents. The obtained recovery values were higher than those recorded for the pure carbon materials and halloysite nanotubes, exceeding 80% for some pesticides. With acetone as the eluent, the highest recoveries were observed for Florisil (81% chlorpyrifos), active carbon (88% ethoprophos and 85% disulfoton), and C-18 (96% ethoprophos, 92% disulfoton, and 96% parathion-methyl). For the ethyl acetate-n-hexane mixture, the highest values were achieved by Florisil (82% ethoprophos and 88% fenchlorphos), active carbon (96% disulfoton), and C-18 (82% fenchlorphos). Nevertheless, these commercial sorbents did not outperform the mineral–carbon composites.

To evaluate the applicability of the synthesized mineral–carbon composites, their extraction performance was compared with other reported sorbents used for the determination of organophosphorus pesticides. As discussed previously, various materials have been investigated for this purpose. For instance, the application of dual-template molecularly imprinted polymers (MIPs) for the extraction of twelve OPPs resulted in recoveries ranging from 81.5% to 113.4% [25]. Similarly, a graphene aerogel-supported SPE method enabled the detection of OPPs with recovery values between 91.2% and 103.7% [20]. Furthermore, magnetic graphene oxide-covalent organic framework (MGO-COF) composites utilized in SPE allowed for pesticide detection with recoveries of 91.3–109% [34].

In the present study, the optimized SPE procedure utilizing the novel composites (such as D-HNT and K-HNT) and an ethyl acetate-n-hexane (1:1, *v*/*v*) eluent yielded high recoveries exceeding 80% for the majority of the targeted pesticides. For example, the D-HNT composite achieved recoveries reaching up to 118%, while the K-HNT composite reached up to 108%. These results demonstrate that the extraction efficiency of the proposed materials is comparable to state-of-the-art sorbents, such as MIPs and graphene-based materials. Considering that the composites synthesized in this work are produced from sustainable, low-cost, and waste-derived precursors (casein, dextrin, halloysite nanotubes, and pyrolyzed sawdust), they represent an economically advantageous alternative for environmental water monitoring.

## 3. Materials and Methods

### 3.1. Reagents, Materials, and Instruments

The standard solution of 8 organophosphorus pesticides (azinphos methyl, chlorpyrifos, dichlorvos, disulfoton, ethoprofos, fenchlorfos, parathion-methyl, and prothiofos), each at a concentration of 2000 µg/mL in hexane:acetone (9:1), was purchased from Supelco by Sigma-Aldrich (St. Louis, MO, USA). Methanol, acetone, n-hexane, ethyl acetate, and dichloromethane (≥99% purity, for GC analysis) were purchased from Pestinorm VWR International (Radnor, PA, USA). Disposable extraction columns (1 mL) with PTFE frits and an SPE vacuum manifold were obtained from J.T. Baker (Phillipsburg, NJ, USA). Determination of OPs was carried out using gas chromatography coupled with a mass spectrometry system GCMS-TQ8030 by Shimadzu Corporation (Kyoto, Japan). To produce carbon–mineral composites, casein and dextrin were purchased from Pol-Aura (Morąg, Poland), and halloysite nanoclay was obtained from Sigma-Aldrich (Darmstadt, Germany).

### 3.2. Mineral–Carbon Composites Synthesis

#### 3.2.1. K-HNT

Casein was mixed with water and halloysite nanotubes (HNTs) at mass ratios of (1:3) and (1:1), respectively. The resulting mixture was transferred into a quartz vessel and dried for 1 h at 105 °C to remove water. After drying, the sample was subjected to heating in a furnace under a nitrogen atmosphere (flow rate 33–35 mL/min), with a heating rate of 5 °C/min, until reaching the target temperature of 800 °C. The sample was maintained at this temperature for 1 h.

#### 3.2.2. K-C-HNT

Pine sawdust (mean particle length ~5 mm) was loaded into a quartz vessel and thermally treated in a furnace under a nitrogen atmosphere. Sample was heated from room temperature to 800 °C at 5 °C/min and held at 800 °C for 1 h. After cooling to room temperature, the resulting carbon material was recovered. Casein was dissolved in deionized water at a mass ratio of 1:3 (casein:water). The mixture was combined with halloysite nanotubes (HNTs) and previously pyrolyzed pine sawdust (as an additional carbon source) in a mass ratio of 2:1:1 (casein:HNT:pyrolyzed sawdust). The final pyrolysis method was carried out using the same protocol applied to the K-HNT sample.

#### 3.2.3. D-HNT

Dextrin was mixed with water at a mass ratio of (1:1) and heated in a water bath at approximately 60–70 °C until completely dissolved. The solution was then cooled, and the appropriate amount of halloysite nanotubes (HNTs) was added at a mass ratio of (1:1) relative to dextrin, followed by thorough mixing. The resulting mixture was transferred into a quartz vessel and dried for 1 h at 105 °C to remove water. After drying, the sample was subjected to heating in a furnace under a nitrogen atmosphere (flow rate 33–35 mL/min), with a heating rate of 5 °C/min, until reaching the target temperature of 800 °C. The sample was maintained at this temperature for 1 h.

#### 3.2.4. D-C-HNT

Pine sawdust (average particle length—5 mm) was placed in a quartz vessel and heated in a nitrogen atmosphere. Sample was heated from room temperature to 800 °C at 5 °C/min and maintained at 800 °C for 1 h. After cooling to ambient temperature, the resulting carbon material was collected. Dextrin was dissolved in deionized water at a mass ratio of 1:1 (dextrin:water). The solution was mixed with halloysite nanotubes (HNTs) and previously pyrolyzed pine sawdust (additional carbon source) in a mass ratio of 2:1:1 (dextrin:HNT:pyrolyzed sawdust). Final pyrolysis was performed following the same protocol used for the D-HNT sample. Table 7 shows the materials used in the synthesis of the mineral–carbon composites, including the mass ratios of the individual components.

### 3.3. Structural Properties Investigation

The standard parameters of the porous structure, including specific surface area, pore volume, and mesoporosity, were determined using low-temperature (−196 °C) nitrogen adsorption–desorption isotherms through a volumetric analyzer (ASAP 2020, Micromeritics, Norcross, GA, USA). The specific surface area (S_BET_) was calculated within the relative pressure range of 0.05–0.2, considering the surface area occupied by a single nitrogen molecule in a monolayer (0.162 nm^2^). The total pore volume (V_t_) was determined from the isotherm point corresponding to the relative pressure p/p_0_ = 0.99.

### 3.4. FT-IR Spectroscopy Measurements

FT-IR spectra were collected in the range of 4000–650 cm^−1^ using a Perkin Elmer Spectrum 400 FT-IR/FT-NIR spectrometer (Shelton, CT, USA) fitted with a diamond attenuated total reflectance (ATR) accessory. Prior to analysis, the samples were dried and homogenized by grinding in an agate mortar. Each spectrum was obtained by averaging 40 scans at a resolution of 4 cm^−1^.

### 3.5. Raman Spectroscopy Measurements

Raman spectra were collected in the spectral range of 2500–450 cm^−1^ using a Perkin Elmer Raman Station 400 spectrometer (Shelton, CT, USA) equipped with a CCD detector and a 785 nm diode laser (350 mW). The samples were dried before analysis. For each sample, five scans were recorded with an acquisition time of 20 s per scan and a spectral resolution of 1 cm^−1^.

### 3.6. SEM/EDS Characterization

SEM/EDS analysis of the investigated materials was conducted using a high-resolution scanning electron microscope, the SUPRA 35 (ZEISS, Oberkochen, Germany). The instrument was equipped with an In-Lens secondary electron (SE) detector and a backscattered electron (BSE) detector, enabling detailed examination of surface morphology and compositional contrast. Elemental and microstructural characterization were further supported by an integrated TRIDENT XM4 system (EDAX, Mahwah, NJ, USA), which combines energy-dispersive X-ray spectroscopy (EDS), wavelength-dispersive spectroscopy (WDS), and electron backscatter diffraction (EBSD) modules.

### 3.7. The Elaborated Procedure of Solid-Phase Extraction

Solid-phase extraction (SPE) was performed using columns filled with 50 mg of the selected mineral–carbon composite with a grain size of 0.6 mm. Each sorbent was packed in a 1 cm^3^ glass cartridge between two polytetrafluoroethylene frits. The columns were placed in the extraction chamber, where the pressure was maintained between −200 and −400 mbar and conditioned with 2 × 1 cm^3^ of methanol followed by 2 × 1 cm^3^ of distilled water. In the next step, 10 cm^3^ of the organophosphorus pesticides solution (where the initial concentration of each individual pesticide was maintained constant at 50 µg/L) was passed through the glass cartridge, filled with mineral–carbon composite, at the flow rate of 1 cm^3^ min^−1^. After sample application, the columns were dried for 15 min under vacuum. Analytes were subsequently eluted with 1 cm^3^ of solvent, using one of the following eluents: acetone, ethyl acetate-n-hexane (1:1, *v*/*v*), or dichloromethane. Finally, the recovered eluate (approximately 1 mL) was collected, and a 200 µL analytical aliquot was transferred into a GC chromatographic vial equipped with a micro-insert for the 2 µL injection during GC-MS/MS analysis.

All SPE procedures were performed in triplicate (n = 3). To ensure no background contamination from the composites, blank extractions using distilled water were performed. The extraction efficiency (Recovery, %) was calculated based on the total mass of the pesticide before and after extraction, using the following Equation (3):

(3) $$Recovery\left(\%\right)=\frac{C_{eluate}\cdot V_{eluate}}{C_{initial}\cdot V_{sample}}\cdot 100\%$$

where C_eluate_ is the concentration of the pesticide in the eluate, V_eluate_ is the volume of the elution solvent (1 mL), C_initial_ is the initial concentration of the pesticide in the water sample (50 µg/L), and V_sample_ is the volume of the loaded sample (10 mL).

### 3.8. GC-MS Analysis Conditions

GC-MS/MS analyses were carried out using a TQ8040NX model Shimadzu, Corporation (Kyoto, Japan). The injector port temperature was set to 250 °C and operated in splitless mode. Helium (99.999% purity) was used as the carrier gas at a constant linear velocity of 44.7 cm·s^−1^. A sample volume of 2 μL was injected. The ion source and transfer line temperatures were maintained at 230 °C and 250 °C, respectively. Target analytes were quantified in Multiple Reaction Monitoring (MRM) mode, with the target and reference ions and collision energies provided in Table A1 (Appendix A). Chromatographic separation was achieved on an Rtx-5MS capillary column (30 m length, 0.25 mm I.D., df = 0.25 µm). The oven temperature program started at 80 °C (held for 1 min), followed by a ramp of 20 °C·min^−1^ to 280 °C, where it was held for 3 min. The total time of the GC-MS/MS analysis was 14 min. Calibration standards were prepared in the concentration range of 10–200 μg·L^−1^ by dilution of a pesticide mixed standard solution. Linearity, regression coefficients, limits of detection (LODs), and limits of quantification (LOQs) are presented in Table A2 and Table A3 (Appendix A).

## 4. Conclusions

In this study, the structural properties and application potential of mineral–carbon composites (K-HNT, K-C-HNT, D-HNT, and D-C-HNT) were evaluated. Porous structure analyses revealed that dextrin-based composites exhibited higher specific surface area (218.25 m^2^ g^−1^ for D-C-HNT) and a more microporous character compared to casein-based materials (45 m^2^ g^−1^ for K-HNT). Spectroscopic and microscopic investigations confirmed the coverage of halloysite nanotubes with an amorphous carbon phase. Solid-phase extraction (SPE) combined with GC-MS/MS analysis proved to be an effective tool for determining trace amounts of six selected organophosphorus pesticides in an aqueous environment. The optimization demonstrated that an ethyl acetate/n-hexane mixture (1:1, *v*/*v*) and sorbent masses of 50 and 75 mg were most effective, yielding recoveries exceeding 80%. Notably, the synthesized composites outperformed pure precursor materials and demonstrated extraction efficiencies comparable to commercial sorbents, such as C-18, Florisil, and activated carbon. Stability tests proved that the sorbents can be reused multiple times with only a moderate decrease in recoveries (e.g., from 100–118% in the first cycle to an acceptable range of 72–88% in the third cycle for the D-HNT material), aligning with the principles of green chemistry.

## Figures and Tables

**Table 1:** Porous structure parameters of the investigated mineral–carbon composites and the precursor materials.

Mineral–Carbon Composite	S_BET_ (m^2^ g^−1^)	V_t_ (cm^3^ g^−1^)	V_micro_ (cm^3^ g^−1^)	V_meso_ (cm^3^ g^−1^)	Mesoporosity (%)
K-HNT	44.2	0.1105	0.0075	0.1030	93.21
K-C-HNT	32.8	0.0357	0.0094	0.0263	73.67
D-HNT	115.6	0.0862	0.0332	0.0530	61.48
D-C-HNT	218.3	0.1245	0.0796	0.0449	36.06
Casein	1.9	0.0038	-	0.0038	100.00
Dextrin	308.0	0.1484	0.1294	0.0190	12.80
HNT	62.7	0.1958	0.0081	0.1877	95.86
Pyrolyzed sawdust	351.1	0.1686	0.1462	0.0224	13.29

S_BET_—specific surface area BET, V_t_—total pore volume calculated from single point on nitrogen adsorption isotherm at p/p_0_ ≈ 0.99, V_micro_—micropores volume (pores < 2 nm) obtained from DFT PSD, V_meso_—mesopores volume (pores 2–50 nm) obtained from DFT PSD, Mesoporosity (%)—volume fraction of mesopores (V_meso_) relative to the total pore volume (V_t_) of the composite, calculated using the following formula: Mesoporosity (%) = (V_meso_/V_total_)·100%.

**Table 2:** The values of the R parameter and crystallinity coefficients for the studied mineral–carbon composites and carbon materials.

Material	R Value	Crystallinity Coefficient K, %
K-HNT	0.841	54.313
K-C-HNT	0.842	54.301
D-HNT	0.834	54.515
D-C-HNT	0.834	54.512
casein	0.848	54.100
dextrin	0.845	54.201
pyrolyzed sawdust	0.834	54.511

**Table 3:** Comparison of pure materials for SPE of selected OPs, sorbent mass: 50 mg, 10 cm^3^ of sample loading volume, concentration of pesticides: 50 μg dm^−3^, acetone as the eluent.

Sorbent Name	Recovery [%]					
	Ethoprophos	Disulfoton	Parathion-Methyl	Fenchlorphos	Chlorpyrifos	Prothiofos
Casein	43 ± 1.2	60 ± 2.1	48 ± 0.6	53 ± 1.3	40 ± 1.2	22 ± 0.5
Dextrin	13 ± 0.4	72 ± 2.5	52 ± 0.6	67 ± 1.6	67 ± 2.1	34 ± 0.7
HNT	27 ± 0.7	31 ± 1.1	11 ± 0.1	28 ± 0.7	40 ± 1.2	21 ± 0.5
Pyrolyzed sawdust	62 ± 1.7	58 ± 2.0	16 ± 0.2	46 ± 1.1	46 ± 1.4	16 ± 0.4

**Table 4:** Comparison of pure materials for SPE of selected OPs, sorbent mass: 50 mg, 10 cm^3^ of sample loading volume, concentration of pesticides: 50 μg dm^−3^, ethyl acetate-n-hexane (1:1) as the eluent.

Sorbent Name	Recovery [%]					
	Ethoprophos	Disulfoton	Parathion-Methyl	Fenchlorphos	Chlorpyrifos	Prothiofos
Casein	72 ± 1.9	66 ± 2.3	34 ± 0.4	58 ± 1.4	62 ± 1.9	50 ± 1.1
Dextrin	32 ± 0.9	64 ± 2.2	30 ± 0.4	64 ± 1.5	70 ± 2.2	52 ± 1.1
HNT	11 ± 0.3	4 ± 0.1	26 ± 0.3	26 ± 0.6	32 ± 1.0	60 ± 1.3
Pyrolyzed sawdust	62 ± 1.7	24 ± 0.8	81 ± 1.0	22 ± 0.5	28 ± 0.9	74 ± 1.6

**Table 5:** Comparison of commercial materials for SPE of selected OPs sorbent mass: 50 mg, 10 cm^3^ of sample loading volume, concentration of pesticides: 50 μg dm^−3^, acetone as the eluent.

Sorbent Name	Recovery [%]					
	Ethoprophos	Disulfoton	Parathion-Methyl	Fenchlorphos	Chlorpyrifos	Prothiofos
Florisil	47 ± 1.3	6 ± 0.2	26 ± 0.3	55 ± 1.3	81 ± 2.5	69 ± 1.5
Active carbon	88 ± 2.4	85 ± 3.0	14 ± 0.2	45 ± 1.1	56 ± 1.7	45 ± 1.0
C-18	96 ± 2.6	92 ± 3.2	96 ± 1.2	47 ± 1.1	58 ± 1.8	51 ± 1.1

**Table 6:** Comparison of commercial materials for SPE of selected OPs sorbent mass: 50 mg, 10 cm^3^ of sample loading volume, concentration of pesticides: 50 μg dm^−3^, ethyl acetate-n-hexane (1:1) as the eluent.

Sorbent Name	Recovery [%]					
	Ethoprophos	Disulfoton	Parathion-Methyl	Fenchlorphos	Chlorpyrifos	Prothiofos
Florisil	82 ± 2.2	19 ± 0.7	61 ± 0.7	88 ± 2.1	77 ± 2.4	73 ± 1.6
Active carbon	69 ± 1.9	96 ± 3.4	43 ± 0.5	73 ± 1.8	67 ± 2.1	69 ± 1.5
C-18	33 ± 0.9	73 ± 2.6	22 ± 0.3	82 ± 2.0	77 ± 2.4	71 ± 1.6

**Table 7:** Materials for the synthesis of mineral–carbon composites.

Mineral–Carbon Composite	Materials	Mass Ratio (*w*/*w*)
K-HNT	casein–halloysite nanotubes	1:1
K-C-HNT	casein–pyrolyzed sawdust–halloysite nanotubes	2:1:1
D-HNT	dextrin–halloysite nanotubes	1:1
D-C-HNT	dextrin–pyrolyzed sawdust–halloysite nanotubes	2:1:1

## Data Availability

The data generated and/or analyzed during this study are available from the corresponding author upon reasonable request.

## Appendix A

### Appendix A.1

**Table A1:** MRM transition.

Compound	Qualitative Transition		Qualitative Transition 1		Qualitative Transition 2	
	Precursor > Product	CE (V)	Precursor > Product	CE (V)	Precursor > Product	CE (V)
Ethoprophos	200.00 > 158.00	6	157.95 > 114.00	9	157.95 > 97.00	18
Disulfoton	88.00 > 60.00	9	89.05 > 61.00	9	88.00 > 73.00	12
Methyl parathion	263.00 > 109.10	12	125.00 > 79.00	9	109.00 > 79.00	9
Fenchlorphos	284.90 > 269.80	21	125.00 > 79.00	6	286.90 > 271.80	21
Chlorpyrifos	198.95 > 170.90	15	313.95 > 257.80	15	96.95 > 65.00	18
Prothiofos	112.95 > 94.90	15	266.90 > 238.90	12	308.95 > 238.80	15

### Appendix A.2

**Table A2:** RSD peak area (conc.) n = 5, conc. = 50 uL/L.

Compound	RSD [%]	LOD [ug/L]	LOQ [ug/L]
Ethoprophos	2.7	1.6	5
Disulfoton	3.5	1.6	5
Methyl parathion	1.2	3.3	10
Fenchlorphos	2.4	1.6	5
Chlorpyrifos	3.1	3.3	10
Prothiofos	2.2	3.3	10

### Appendix A.3

**Table A3:** Calibration standards, calibration range, and regression coefficients.

Compound	Calibration Range [ug/L]	Calibration Equation	R^2^
Ethoprophos	5–200	y = 2291.37x + 60,381.50	0.96
Disulfoton	5–200	y = 6076.79 + 197,612.7	0.97
Methyl parathion	10–200	y = 793.96 + 3036.84	0.98
Fenchlorphos	5–200	y = 2651.30 + 83,367.48	0.96
Chlorpyrifos	10–200	y = 1683.48 + 60,285.96	0.98
Prothiofos	10–200	y = 1662.75 + 53,938.35	0.97
